# Supplementary material for: Stochastic parametric skeletal dosimetry model for humans: Pediatric and adult computational skeleton phantoms for internal bone marrow dosimetry
Source: PLoS One. 2025 Jul 3;20(7):e0327479. doi: 10.1371/journal.pone.0327479 (PMC12225816; doi:10.1371/journal.pone.0327479)
Supplement: S1 File — (DOCX) [file pone.0327479.s001.docx]

**Supplementary material S1. Individual variability of the parameters of bone segment phantoms**

Table S1.1 Coefficients of variation (%) of parameters of bone segment phantoms and a range of possible values is specified for *BV/TV* for newborns (0-Y)

| Site | Segment | h | a | b | c | d | Ct.Th | BV/TV | Tb.Th | Tb.Sp |
| --- | --- | --- | --- | --- | --- | --- | --- | --- | --- | --- |
| Clavicle | Acromeon end | 15 | 24 | 49 | 25 | 23 | 24 | 0.15-0.46 | 13 | 25 |
| Clavicle | Shaft | 15 | 25 | 23 |  |  | 25 | 0.15-0.46 | 13 | 25 |
| Clavicle | Sternal end | 15 | 24 | 24 | 25 | 23 | 24 | 0.15-0.46 | 13 | 25 |
| Femur | Distal end | 5 | 9 | 12 | 11 | 11 | 25 | 0.26-0.53 | 15 | 27 |
| Femur | Proximal end | 5 | 9 | 12 | 11 | 11 | 24 | 0.26-0.53 | 15 | 27 |
| Femur | Shaft |  | 11 | 11 |  |  | 24 | 0.26-0.53 | 15 | 27 |
| Hand and foot | Precarpal |  | 14 | 11 | 14 |  | 25 | 0.05-0.72 | 27 | 52 |
| Hand and foot | Tube bones | 43 | 42 | 42 |  |  | 25 | 0.05-0.72 | 27 | 52 |
| Humerus | Distal end | 10 | 13 | 12 | 12 | 11 | 19 | 0.16-0.48 | 51 | 11 |
| Humerus | Proximal end | 10 | 12 | 12 | 12 | 11 | 18 | 0.16-0.48 | 51 | 11 |
| Humerus | Shaft |  | 12 | 12 |  |  | 15 | 0.16-0.48 | 51 | 11 |
| Pelvis | Ilium part 1 | 23 | 3 | 3 |  |  | 33  47 | 0.13-0.56 | 14 | 32 |
| Pelvis | Ilium part 2 | 23 | 3 | 3 |  |  | 25 | 0.13-0.56 | 14 | 32 |
| Pelvis | Ischium | 16 | 11 | 8 |  |  | 9 | 0.13-0.56 | 14 | 32 |
| Pelvis | Pubis | 13 | 16 | 16 |  |  | 9 | 0.13-0.56 | 14 | 32 |
| Radius and ulna | End | 6 | 7 | 7 | 8 | 8 | 29 | 0.11-0.31 | 25 | 24 |
| Radius and ulna | Shaft |  | 8 | 8 |  |  | 13 | 0.11-0.31 | 25 | 24 |
| Ribs | Ribs | 38 |  | 12 |  |  | 37 | 0.1-0.36 | 35 | 10 |
| Sacrum | Body 1 | 21 | 10 | 10 |  |  |  | 0.21-0.94 | 42 | 20 |
| Sacrum | Body 2 | 21 | 10 | 10 |  |  |  | 0.21-0.94 | 42 | 20 |
| Sacrum | Body 3 | 19 | 10 | 9 |  |  |  | 0.21-0.94 | 42 | 20 |
| Sacrum | Body 4 | 21 | 10 | 9 |  |  |  | 0.21-0.94 | 42 | 20 |
| Sacrum | Body 5 | 21 | 10 | 11 |  |  |  | 0.21-0.94 | 42 | 20 |
| Scapula | Acromion | 19 | 14 | 25 |  |  | 13 | 0.12-0.44 | 83 | 50 |
| Scapula | Body | 13 |  |  |  |  | 17 | 0.12-0.44 | 83 | 50 |
| Scapula | Glenoid | 4 | 21 | 18 |  |  | 29 | 0.12-0.44 | 83 | 50 |
| Skull | Flat bones | 25 |  |  |  |  |  | 0.41-0.65 | 32 | 35 |
| Tibia and fibula | Distal end | 9 | 23 | 23 | 28 | 28 | 17 | 0.21-0.57 | 9 | 22 |
| Tibia and fibula | Fibula shaft |  | 7 | 7 |  |  | 14 | 0.24-0.42 | 9 | 22 |
| Tibia and fibula | Proximal end | 9 | 9 | 18 | 28 | 28 | 17 | 0.21-0.57 | 9 | 22 |
| Tibia and fibula | Tibia shaft |  | 28 | 28 |  |  | 14 | 0.21-0.57 | 9 | 22 |
| Vertebra | C-body | 1 | 1 | 1 |  |  |  | 0.46-0.77 | 6 | 20 |
| Vertebra | L-body | 1 | 5 | 1 |  |  |  | 0.21-0.94 | 42 | 20 |
| Vertebra | T-body | 2 | 2 | 2 |  |  |  | 0.21-0.94 | 42 | 20 |

Table S1.2 Coefficients of variation (%) of parameters of bone segment phantoms and a range of possible values is specified for BV/TV for 1-year-old child

| Site | Segment | h | a | b | c | d | Ct.Th | BV/TV | Tb.Th | Tb.Sp |
| --- | --- | --- | --- | --- | --- | --- | --- | --- | --- | --- |
| Clavicle | Acromial end | 11 | 10 | 19 | 10 | 10 | 10 | 0.15-0.46 | 13 | 25 |
| Clavicle | Shaft | 11 | 10 | 10 |  |  | 10 | 0.15-0.46 | 13 | 25 |
| Clavicle | Sternal end | 11 | 10 | 9 | 10 | 10 | 10 | 0.15-0.46 | 13 | 25 |
| Femur | Distal end | 4 | 12 | 8 | 7 | 7 | 17 | 0.12-0.4 | 38 | 20 |
| Femur | Proximal end | 4 | 12 | 8 | 7 | 7 | 17 | 0.12-0.4 | 38 | 20 |
| Femur | Shaft |  | 7 | 7 |  |  | 17 | 0.12-0.4 | 38 | 20 |
| Humerus | Distal end | 13 | 16 | 13 | 13 | 13 | 20 | 0.12-0.4 | 30 | 7 |
| Humerus | Proximal end | 13 | 13 | 13 | 13 | 13 | 20 | 0.12-0.4 | 30 | 7 |
| Humerus | Shaft |  | 13 | 13 |  |  | 19 | 0.12-0.4 | 30 | 7 |
| Pelvis | Ilium acetabular part | 10 | 9 | 30 | 22 | 40 | 30 | 0.18-0.28 | 20 | 23 |
| Pelvis | Ilium flat part 1 | 18 |  |  |  |  | 33  47 | 0.18-0.28 | 20 | 23 |
| Pelvis | Ilium flat part 2 | 18 |  |  |  |  | 30 | 0.18-0.28 | 20 | 23 |
| Pelvis | Ischium acetabular part | 15 | 15 | 30 | 15 |  | 3 | 0.18-0.28 | 20 | 23 |
| Pelvis | Ischium tuberosity | 15 | 15 | 15 |  |  | 3 | 0.18-0.28 | 20 | 23 |
| Pelvis | Pubic ramus superior | 15 | 11 | 11 |  |  | 30 | 0.18-0.28 | 20 | 23 |
| Pelvis | Pubis acetabular part | 15 | 3 | 7 | 11 | 11 | 30 | 0.18-0.28 | 20 | 23 |
| Radius and ulna | End | 6 | 6 | 6 | 6 | 6 | 29 | 0.08-0.31 | 15 | 16 |
| Radius and ulna | Shaft |  | 6 | 6 |  |  | 13 | 0.08-0.31 | 15 | 16 |
| Ribs | Ribs | 32 |  | 35 |  |  | 33 | 0.13-0.63 | 34 | 14 |
| Sacrum | Ala 1 | 20 | 10 | 10 |  |  |  | 0.07-0.26 | 42 | 20 |
| Sacrum | Ala 2 | 20 | 10 | 10 |  |  |  | 0.07-0.26 | 42 | 20 |
| Sacrum | Ala 3 | 20 | 10 | 10 |  |  |  | 0.07-0.26 | 42 | 20 |
| Sacrum | Ala 4 | 20 | 9 | 10 |  |  |  | 0.07-0.26 | 42 | 20 |
| Sacrum | Body | 20 | 10 | 10 |  |  |  | 0.07-0.26 | 42 | 20 |
| Sacrum | Body | 20 | 10 | 10 |  |  |  | 0.07-0.26 | 42 | 20 |
| Sacrum | Body | 20 | 10 | 10 |  |  |  | 0.07-0.26 | 42 | 20 |
| Sacrum | Body | 20 | 10 | 10 |  |  |  | 0.07-0.26 | 42 | 20 |
| Sacrum | Body | 20 | 10 | 10 |  |  |  | 0.07-0.26 | 42 | 20 |
| Scapula | Acromion | 19 | 41 | 42 |  |  | 13 | 0.09-0.47 | 52 | 23 |
| Scapula | Body | 13 |  |  |  |  | 13 | 0.09-0.47 | 52 | 23 |
| Scapula | Glenoid | 26 | 18 | 29 |  |  | 29 | 0.09-0.47 | 52 | 23 |
| Skull | Flat bones | 30 |  |  |  |  | 29 | 0.33-0.71 | 32 | 35 |
| Tibia and fibula | Distal end | 6 | 23 | 23 | 13 | 13 | 14 | 0.14-0.29 | 9 | 14 |
| Tibia and fibula | Fibula shaft |  | 11 | 11 |  |  | 17 | 0.14-0.29 | 9 | 11 |
| Tibia and fibula | Proximal end | 6 | 12 | 18 | 13 | 13 | 14 | 0.14-0.29 | 9 | 14 |
| Tibia and fibula | Tibia shaft |  | 13 | 13 |  |  | 9 | 0.14-0.29 | 9 | 14 |
| Vertebra | Cervical vertebra body | 9 | 7 | 7 |  |  |  | 0.12-0.33 | 13 | 20 |
| Vertebra | Lumbar vertebra body | 16 | 3 | 7 |  |  |  | 0.07-0.27 | 42 | 20 |
| Vertebra | Thoracic vertebra body | 15 | 23 | 13 |  |  |  | 0.07-0.27 | 42 | 20 |

Table S1.3 Coefficients of variation (%) of parameters of bone segment phantoms and a range of possible values is specified for for 5- year-old child

| Site | Segment | h | a | b | c | d | Ct.Th | BV/TV | Tb.Th | Tb.Sp |
| --- | --- | --- | --- | --- | --- | --- | --- | --- | --- | --- |
| Clavicle | Acromial end | 13 | 10 | 18 | 9 | 10 | 10 | 0.14-0.56 | 13 | 25 |
| Clavicle | Shaft |  | 9 | 10 |  |  | 9 | 0.1-0.23 | 32 | 25 |
| Clavicle | Sternal end | 13 | 10 | 9 | 9 | 10 | 10 | 0.14-0.56 | 13 | 25 |
| Femur | Distal end | 4 | 6 | 7 | 6 | 6 | 7 | 0.17-0.39 | 22 | 14 |
| Femur | Lower proximal end | 4 | 30 | 30 |  |  | 14 | 0.23-0.52 | 22 | 14 |
| Femur | Shaft |  | 6 | 6 |  |  | 8 | 0.17-0.39 | 22 | 14 |
| Femur | Upper proximal end | 4 | 30 | 30 |  |  | 14 | 0.23-0.52 | 22 | 14 |
| Humerus | Distal end | 4 | 7 | 3 | 3 | 3 | 19 | 0.09-0.48 | 13 | 32 |
| Humerus | Proximal end | 4 | 5 | 5 | 5 | 5 | 18 | 0.09-0.48 | 13 | 32 |
| Humerus | Shaft |  | 3 | 3 |  |  | 20 | 0.09-0.48 | 13 | 32 |
| Pelvis | Ilium acetabular part | 8 | 10 | 30 | 30 | 30 | 20 | 0.2-0.31 | 10 | 23 |
| Pelvis | Ilium flat part 1 | 13 |  |  |  |  | 33  20 | 0.2-0.31 | 10 | 23 |
| Pelvis | Ilium flat part 2 | 13 |  |  |  |  | 20 | 0.2-0.31 | 10 | 23 |
| Pelvis | Ischial ramus inferior | 15 | 20 | 20 |  |  | 30 | 0.2-0.31 | 10 | 23 |
| Pelvis | Ischium acetabular part | 15 | 15 | 15 | 15 |  | 30 | 0.2-0.31 | 10 | 23 |
| Pelvis | Ischium tuberosity | 15 | 15 | 15 |  |  | 30 | 0.2-0.31 | 10 | 23 |
| Pelvis | Pubis acetabular part | 15 | 20 | 20 | 11 | 20 | 30 | 0.2-0.31 | 10 | 23 |
| Pelvis | Pubis ramus inferior | 15 | 20 | 20 |  |  | 30 | 0.2-0.31 | 10 | 23 |
| Pelvis | Pubis ramus superior | 15 | 11 | 20 |  |  | 30 | 0.2-0.31 | 10 | 23 |
| Radius and ulna | End | 5 | 6 | 5 | 5 | 5 | 29 | 0.06-0.26 | 13 | 14 |
| Radius and ulna | Shaft |  | 25 | 17 |  |  | 12 | 0.06-0.26 | 13 | 14 |
| Ribs | Ribs | 32 |  | 35 |  |  | 33 | 0.1-0.37 | 34 | 14 |
| Sacrum | Body-ala 1 | 20 | 20 | 6 |  |  | 35 | 0.07-0.26 | 42 | 20 |
| Sacrum | Body-ala 2 | 20 | 20 | 10 |  |  | 35 | 0.07-0.26 | 42 | 20 |
| Sacrum | Body-ala 3 | 20 | 20 | 10 |  |  | 35 | 0.07-0.26 | 42 | 20 |
| Sacrum | Body-ala 4 | 20 | 20 | 9 |  |  | 35 | 0.07-0.26 | 42 | 20 |
| Sacrum | Body-ala 5 | 20 | 20 | 9 |  |  | 35 | 0.07-0.26 | 42 | 20 |
| Scapula | Acromion | 18 | 12 | 12 |  |  | 13 | 0.09-0.47 | 42 | 23 |
| Scapula | Glenoid | 8 | 11 | 7 |  |  | 28 | 0.09-0.47 | 42 | 23 |
| Scapula | Lateral margin |  | 6 | 12 |  |  | 13 | 0.09-0.47 | 42 | 23 |
| Skull | Flat bones | 26 |  |  |  |  | 26 | 0.41-0.65 | 32 | 35 |
| Sternum | Sternum | 13 |  |  |  |  | 19 | 0.07-0.23 | 30 | 6 |
| Tibia and fibula | Distal end | 5 | 22 | 23 | 4 | 4 | 18 | 0.18-0.35 | 13 | 11 |
| Tibia and fibula | Fibula body |  | 6 | 6 |  |  | 20 | 0.2-0.31 | 13 | 11 |
| Tibia and fibula | Proximal end | 5 | 6 | 20 | 4 | 4 | 18 | 0.18-0.35 | 13 | 11 |
| Tibia and fibula | Tibia shaft |  | 4 | 4 |  |  | 17 | 0.18-0.35 | 13 | 11 |
| Vertebra | C-body | 8 | 12 | 12 |  |  | 7 | 0.12-0.35 | 14 | 20 |
| Vertebra | L-body | 11 | 13 | 12 |  |  | 25 | 0.07-0.26 | 42 | 20 |
| Vertebra | L-spinous proc | 20 | 20 | 20 |  |  | 25 | 0.07-0.26 | 42 | 20 |
| Vertebra | L-transverse proc | 13 | 12 | 12 |  |  | 25 | 0.07-0.26 | 42 | 20 |
| Vertebra | T-body | 17 | 20 | 20 |  |  | 25 | 0.07-0.26 | 42 | 20 |
| Vertebra | T-spinous proc | 21 | 21 | 21 |  |  | 25 | 0.07-0.26 | 42 | 20 |
| Vertebra | T-transverse proc | 19 | 19 | 19 |  |  | 25 | 0.07-0.26 | 42 | 20 |

Table S1.4 Coefficients of variation (%) of parameters of bone segment phantoms and a range of possible values is specified for 10- year-old child

| Site | Segment | h | a | b | c | d | Ct.Th | BV/TV | Tb.Th | Tb.Sp |
| --- | --- | --- | --- | --- | --- | --- | --- | --- | --- | --- |
| Clavicle | Acromial end | 7 | 11 | 23 | 12 | 10 | 26 | 0.15-0.46 | 13 | 25 |
| Clavicle | Body |  | 12 | 10 |  |  | 26 | 0.1-0.23 | 32 | 25 |
| Clavicle | Sternal end | 7 | 11 | 11 | 12 | 10 | 26 | 0.15-0.46 | 13 | 25 |
| Femur | Distal end | 5 | 7 | 10 | 9 | 9 | 14 | 0.17-0.4 | 22 | 14 |
| Femur | Lower proximal end |  | 7 | 7 |  |  | 12 | 0.22-0.53 | 22 | 14 |
| Femur | Upper proximal end |  | 7 | 7 |  |  | 17 | 0.22-0.53 | 22 | 14 |
| Humery | Distal end | 4 | 9 | 4 | 4 | 4 | 10 | 0.13-0.37 | 13 | 32 |
| Humery | Proximal end | 4 | 4 | 5 | 4 | 4 | 16 | 0.13-0.37 | 13 | 32 |
| Pelvis | Ilium acetabular part | 7 | 7 | 22 | 3 | 30 | 17 | 0.2-0.31 | 10 | 15 |
| Pelvis | Ilium flat part 1 | 22 |  |  |  |  | 33  19 | 0.2-0.31 | 10 | 15 |
| Pelvis | Ilium flat part 2 | 22 |  |  |  |  | 19 | 0.2-0.31 | 10 | 15 |
| Pelvis | Ischial ramus inferior | 15 | 10 | 10 |  |  | 30 | 0.2-0.31 | 10 | 12 |
| Pelvis | Ischium acetabular part | 15 | 15 | 7 | 7 |  | 30 | 0.2-0.31 | 10 | 12 |
| Pelvis | Ischium tuberosity | 15 | 15 | 15 |  |  | 30 | 0.2-0.31 | 10 | 12 |
| Pelvis | Pubis acetabular part | 15 | 9 | 10 | 9 | 10 | 30 | 0.2-0.31 | 10 | 12 |
| Pelvis | Pubis ramus inferior | 15 | 10 | 10 |  |  | 30 | 0.2-0.31 | 10 | 12 |
| Pelvis | Pubis ramus superior | 15 | 9 | 10 |  |  | 30 | 0.2-0.31 | 10 | 12 |
| Ribs | Ribs | 16 |  | 10 |  |  | 20 | 0.1-0.37 | 34 | 14 |
| Sacrum | Body-ala 1 | 20 | 20 | 10 |  |  | 34 | 0.07-0.26 | 17 | 24 |
| Sacrum | Body-ala 2 | 20 | 20 | 10 |  |  | 34 | 0.07-0.26 | 17 | 24 |
| Sacrum | Body-ala 3 | 20 | 20 | 10 |  |  | 34 | 0.07-0.26 | 17 | 24 |
| Sacrum | Body-ala 4 | 20 | 20 | 10 |  |  | 34 | 0.07-0.26 | 17 | 24 |
| Sacrum | Body-ala 5 | 20 | 20 | 10 |  |  | 34 | 0.07-0.26 | 17 | 24 |
| Scapula | Acromion | 18 | 8 | 8 |  |  | 13 | 0.06-0.38 | 42 | 23 |
| Scapula | Glenoid | 5 | 5 | 7 |  |  | 28 | 0.06-0.38 | 42 | 23 |
| Scapula | Lateral margin |  | 3 | 12 |  |  | 13 | 0.06-0.38 | 42 | 23 |
| Skull | Flat bones | 18 |  |  |  |  | 18 | 0.41-0.65 | 32 | 35 |
| Sternum | Sternum | 15 |  |  |  |  | 62 | 0.07-0.23 | 27 | 6 |
| Tibia and fibula | Distal end | 6 | 12 | 12 | 6 | 6 | 11 | 0.18-0.34 | 13 | 11 |
| Tibia and fibula | Fibula ends |  | 6 | 6 |  |  | 12 | 0.2-0.31 | 13 | 11 |
| Tibia and fibula | Proximal end | 6 | 7 | 20 | 6 | 6 | 11 | 0.18-0.34 | 13 | 11 |
| Vertebra | C-body | 12 | 13 | 7 |  |  | 7 | 0.12-0.35 | 14 | 24 |
| Vertebra | L-body | 18 | 21 | 21 |  |  | 25 | 0.07-0.26 | 17 | 24 |
| Vertebra | L-spinous proc | 20 | 20 | 20 |  |  | 25 | 0.07-0.26 | 17 | 24 |
| Vertebra | L-transverse proc | 20 | 20 | 20 |  |  | 25 | 0.07-0.26 | 17 | 24 |
| Vertebra | T-body | 17 | 24 | 21 |  |  | 25 | 0.07-0.26 | 17 | 24 |
| Vertebra | T-spinous proc | 21 | 21 | 21 |  |  | 25 | 0.07-0.26 | 17 | 24 |
| Vertebra | T-transverse proc | 21 | 21 | 21 |  |  | 25 | 0.07-0.26 | 17 | 24 |

Table S1.5 Coefficients of variation (%) of parameters of bone segment phantoms and a range of possible values is specified for 15- year-old male

| Site | Segment | h | a | b | c | d | Ct.Th | BV/TV | Tb.Th | Tb.Sp |
| --- | --- | --- | --- | --- | --- | --- | --- | --- | --- | --- |
| Clavicle | Acromial end | 5 | 9 | 29 | 17 | 8 | 26 | 0.14-0.56 | 13 | 25 |
| Clavicle | Shaft |  | 17 | 8 |  |  | 2 | 0.1-0.23 | 32 | 25 |
| Clavicle | Sternal end | 5 | 15 | 13 | 17 | 8 | 26 | 0.14-0.56 | 13 | 25 |
| Femur | Neck | 5 | 14 | 13 |  |  | 19 | 0.23-0.51 | 13 | 14 |
| Femur | Trochanter area | 4 | 6 | 6 | 7 | 7 | 15 | 0.15-0.43 | 22 | 14 |
| Humerus | Proximal end | 12 | 5 | 5 | 4 | 4 | 16 | 0.11-0.42 | 13 | 32 |
| Pelvis | Acetabulum | 10 | 10 | 20 |  |  | 30  30 | 0.2-0.31 | 10 | 15 |
| Pelvis | Iliac ala | 31 |  |  |  |  | 30 | 0.2-0.31 | 10 | 15 |
| Pelvis | Iliac crest | 15 |  | 9 |  |  | 30 | 0.2-0.31 | 10 | 15 |
| Pelvis | Iliac dorsal seg. | 16 |  |  |  |  | 30 | 0.2-0.31 | 10 | 15 |
| Pelvis | Ischium ramus |  | 9 | 8 |  |  | 30 | 0.2-0.31 | 10 | 9 |
| Pelvis | Pubis ramus inf. | 17 | 25 | 23 | 23 | 36 | 30 | 0.2-0.31 | 10 | 9 |
| Pelvis | Pubis ramus superior (low) | 19 | 20 | 20 |  |  | 30  12 | 0.2-0.31 | 10 | 9 |
| Pelvis | Pubis ramus superior (upper) | 8 | 20 | 20 |  |  | 30  12 | 0.2-0.31 | 10 | 9 |
| Ribs | 1, 2 | 12 |  | 14 |  |  | 38 | 0.05-0.25 | 12 | 11 |
| Ribs | 11, 12 | 18 |  | 17 |  |  | 38 | 0.05-0.25 | 12 | 11 |
| Ribs | 3, 4, 9, 10 | 8 |  | 14 |  |  | 38 | 0.05-0.25 | 12 | 11 |
| Ribs | 5,6,7,8 | 14 |  | 13 |  |  | 38 | 0.05-0.25 | 12 | 11 |
| Sacrum | Ala 3-4 | 16 | 9 | 15 | 15 |  | 35 | 0.07-0.26 | 17 | 24 |
| Sacrum | Body 1 | 7 | 11 | 10 |  |  | 35 | 0.07-0.26 | 17 | 24 |
| Sacrum | Body 2-3 | 8 | 11 | 9 |  |  | 35 | 0.07-0.26 | 17 | 24 |
| Sacrum | Body 4-5 | 9 | 11 | 13 |  |  | 35 | 0.07-0.26 | 17 | 24 |
| Sacrum | Pedicle 1 | 14 | 15 | 11 |  |  | 35 | 0.07-0.26 | 17 | 24 |
| Sacrum | Pedicle 2 | 14 | 11 | 17 |  |  | 35 | 0.07-0.26 | 17 | 24 |
| Sacrum | Pedicle 3 | 14 | 11 | 14 |  |  | 35 | 0.07-0.26 | 17 | 24 |
| Sacrum | Pedicle 4 | 14 | 11 | 18 |  |  | 35 | 0.07-0.26 | 17 | 24 |
| Sacrum | Sacral ala 1 | 13 | 10 | 13 |  |  | 35 | 0.07-0.26 | 17 | 24 |
| Sacrum | Sacral ala 2 | 15 | 17 | 8 |  |  | 35 | 0.07-0.26 | 17 | 24 |
| Scapula | Acromion | 18 | 4 | 4 |  |  | 13 | 0.09-0.47 | 42 | 23 |
| Scapula | Glenoid | 8 | 11 | 18 |  |  | 28 | 0.09-0.47 | 42 | 23 |
| Scapula | Lateral margin |  | 3 | 12 |  |  | 13 | 0.09-0.47 | 42 | 23 |
| Skull | Flat bones | 12 |  |  |  |  | 12 | 0.41-0.65 | 32 | 35 |
| Sternum | Sternum | 14 |  |  |  |  | 44 | 0.08-0.27 | 33 | 6 |
| Vertebra | C1 mass | 13 | 9 | 9 |  |  | 6 | 0.16-0.28 | 14 | 10 |
| Vertebra | C2-body | 13 | 10 | 3 |  |  | 7 | 0.16-0.28 | 14 | 10 |
| Vertebra | C3-7 body | 11 | 14 | 8 |  |  | 7 | 0.12-0.35 | 14 | 26 |
| Vertebra | L- body | 12 | 9 | 8 |  |  | 25 | 0.07-0.26 | 17 | 24 |
| Vertebra | L- lamina+inf.pr | 10 | 13 | 17 |  |  | 34 | 0.1-0.19 | 13 | 16 |
| Vertebra | L- transverse p. | 10 | 11 | 10 |  |  | 25 | 0.07-0.26 | 17 | 24 |
| Vertebra | L-spinous p. | 10 | 10 | 10 |  |  | 25 | 0.07-0.26 | 17 | 24 |
| Vertebra | L-superior p. | 14 | 13 | 17 |  |  | 34 | 0.1-0.19 | 13 | 16 |
| Vertebra | T- body | 16 | 19 | 20 |  |  | 25 | 0.07-0.26 | 17 | 24 |
| Vertebra | T- lamina+inf. | 12 | 14 | 13 |  |  | 16 | 0.11-0.28 | 15 | 15 |
| Vertebra | T- transverse p. | 18 | 18 | 19 |  |  | 25 | 0.07-0.26 | 17 | 24 |
| Vertebra | T-spinous p. | 18 | 18 | 18 |  |  | 25 | 0.07-0.26 | 17 | 24 |
| Vertebra | T-superior pr. | 12 | 14 | 11 |  |  | 16 | 0.11-0.28 | 15 | 15 |

Table S1.6 Coefficients of variation (%) of parameters of bone segment phantoms and a range of possible values is specified for 15- year-old female

| Site | Segment | h | a | b | c | d | Ct.Th | BV/TV | Tb.Th | Tb.Sp |
| --- | --- | --- | --- | --- | --- | --- | --- | --- | --- | --- |
| Clavicle | Acromial end | 9 | 6 | 32 | 9 | 10 | 26 | 0.14-0.56 | 13 | 25 |
| Clavicle | Shaft |  | 9 | 10 |  |  | 2 | 0.1-0.23 | 32 | 25 |
| Clavicle | Sternal end | 9 | 16 | 14 | 9 | 10 | 26 | 0.14-0.56 | 13 | 25 |
| Femur | Neck | 5 | 10 | 9 |  |  | 14 | 0.23-0.51 | 22 | 14 |
| Femur | Trochanter area | 5 | 7 | 7 | 6 | 6 | 5 | 0.15-0.43 | 22 | 14 |
| Humerus | Proximal end | 10 | 6 | 6 | 4 | 4 | 16 | 0.11-0.42 | 13 | 32 |
| Pelvis | Acetabulum | 10 | 10 | 20 |  |  | 30  30 | 0.2-0.31 | 10 | 15 |
| Pelvis | Iliac ala | 31 |  |  |  |  | 30 | 0.2-0.31 | 10 | 15 |
| Pelvis | Iliac crest | 15 |  | 9 |  |  | 30 | 0.2-0.31 | 10 | 15 |
| Pelvis | Iliac dorsal seg. | 16 |  |  |  |  | 30 | 0.2-0.31 | 10 | 15 |
| Pelvis | Ischium ramus |  | 9 | 8 |  |  | 30 | 0.2-0.31 | 10 | 9 |
| Pelvis | Pubis ramus inf. | 17 | 25 | 23 | 23 | 36 | 30 | 0.2-0.31 | 10 | 9 |
| Pelvis | Pubis ramus superior (Low) | 13 | 7 | 18 |  |  | 30  12 | 0.2-0.31 | 10 | 9 |
| Pelvis | Pubis ramus superior (Upper) | 7 | 18 | 20 |  |  | 30  12 | 0.2-0.31 | 10 | 9 |
| Ribs | 1, 2 | 12 |  | 14 |  |  | 38 | 0.05-0.25 | 12 | 11 |
| Ribs | 11, 12 | 18 |  | 17 |  |  | 38 | 0.05-0.25 | 12 | 11 |
| Ribs | 3, 4, 9, 10 | 8 |  | 14 |  |  | 38 | 0.05-0.25 | 12 | 11 |
| Ribs | 5,6,7,8 | 14 |  | 13 |  |  | 38 | 0.05-0.25 | 12 | 11 |
| Sacrum | Ala 3-4 | 16 | 9 | 15 | 15 |  | 35 | 0.07-0.26 | 17 | 24 |
| Sacrum | Body 1 | 9 | 11 | 12 |  |  | 35 | 0.07-0.26 | 17 | 24 |
| Sacrum | Body 2-3 | 15 | 11 | 13 |  |  | 35 | 0.07-0.26 | 17 | 24 |
| Sacrum | Body 4-5 | 14 | 11 | 12 |  |  | 35 | 0.07-0.26 | 17 | 24 |
| Sacrum | Pedicle 1 | 14 | 15 | 11 |  |  | 35 | 0.07-0.26 | 17 | 24 |
| Sacrum | Pedicle 2 | 14 | 11 | 17 |  |  | 35 | 0.07-0.26 | 17 | 24 |
| Sacrum | Pedicle 3 | 14 | 11 | 14 |  |  | 35 | 0.07-0.26 | 17 | 24 |
| Sacrum | Pedicle 4 | 14 | 11 | 18 |  |  | 35 | 0.07-0.26 | 17 | 24 |
| Sacrum | Sacral ala 1 | 9 | 15 | 8 |  |  | 35 | 0.07-0.26 | 17 | 24 |
| Sacrum | Sacral ala 2 | 9 | 17 | 13 |  |  | 35 | 0.07-0.26 | 17 | 24 |
| Scapula | Acromion | 18 | 4 | 4 |  |  | 13 | 0.09-0.47 | 42 | 23 |
| Scapula | Glenoid | 8 | 11 | 18 |  |  | 28 | 0.09-0.47 | 42 | 23 |
| Scapula | Lateral margin |  | 3 | 12 |  |  | 13 | 0.09-0.47 | 42 | 23 |
| Skull | Flat bones | 12 |  |  |  |  | 12 | 0.41-0.65 | 32 | 35 |
| Sternum | Sternum | 14 |  |  |  |  | 44 | 0.08-0.27 | 33 | 6 |
| Vertebra | C1 mass | 13 | 9 | 9 |  |  | 6 | 0.16-0.28 | 14 | 10 |
| Vertebra | C2-body | 13 | 10 | 3 |  |  | 7 | 0.16-0.28 | 14 | 10 |
| Vertebra | C3-7 body | 11 | 14 | 8 |  |  | 7 | 0.12-0.35 | 14 | 26 |
| Vertebra | L- body | 12 | 9 | 8 |  |  | 25 | 0.07-0.26 | 17 | 24 |
| Vertebra | L- lamina+inf.pr | 10 | 13 | 17 |  |  | 34 | 0.1-0.19 | 13 | 16 |
| Vertebra | L- transverse p. | 10 | 11 | 10 |  |  | 25 | 0.07-0.26 | 17 | 24 |
| Vertebra | L-spinous p. | 10 | 10 | 10 |  |  | 25 | 0.07-0.26 | 17 | 24 |
| Vertebra | L-superior p. | 14 | 13 | 17 |  |  | 34 | 0.1-0.19 | 13 | 16 |
| Vertebra | T- body | 16 | 19 | 20 |  |  | 25 | 0.07-0.26 | 17 | 24 |
| Vertebra | T- lamina+inf. | 12 | 14 | 13 |  |  | 16 | 0.11-0.28 | 15 | 15 |
| Vertebra | T- transverse p. | 18 | 18 | 19 |  |  | 25 | 0.07-0.26 | 17 | 24 |
| Vertebra | T-spinous p. | 18 | 18 | 18 |  |  | 25 | 0.07-0.26 | 17 | 24 |
| Vertebra | T-superior pr. | 12 | 14 | 11 |  |  | 16 | 0.11-0.28 | 15 | 15 |

Table S1.7 Coefficients of variation (%) of parameters of bone segment phantoms and a range of possible values is specified for adult male

| Site | Segment | h | a | b | c | d | Ct.Th | BV/TV | Tb.Th | Tb.Sp |
| --- | --- | --- | --- | --- | --- | --- | --- | --- | --- | --- |
| Clavicle | Acromial end |  | 15 | 12 |  |  | 19 | 0.15-0.46 | 13 | 25 |
| Clavicle | Shaft | 7 | 15 | 12 | 5 | 8 | 2 | 0.08-0.18 | 31 | 25 |
| Clavicle | Sternal end | 7 | 14 | 5 | 9 | 8 | 2 | 0.08-0.18 | 31 | 25 |
| Femur | Neck | 13 | 6 | 4 |  |  | 5 | 0.14-0.22 | 19 | 13 |
| Femur | Trochanter area | 26 | 6 | 6 | 7 | 7 | 15 | 0.08-0.13 | 65 | 20 |
| Humerus | Proximal end | 12 | 5 | 5 | 15 | 15 | 18 | 0.01-0.13 | 18 | 25 |
| Pelvis | Acetabulum | 10 | 10 | 20 |  |  | 30  30 | 0.11-0.25 | 15 | 20 |
| Pelvis | Iliac ala | 31 |  |  |  |  | 30 | 0.11-0.25 | 15 | 20 |
| Pelvis | Iliac crest | 15 |  | 9 |  |  | 30 | 0.11-0.25 | 15 | 20 |
| Pelvis | Iliac dorsal segment | 16 |  |  |  |  | 30 | 0.11-0.25 | 15 | 20 |
| Pelvis | Ischium ramus |  | 9 | 8 |  |  | 30 | 0.23-0.27 | 7 | 40 |
| Pelvis | Pubis ramus inferior | 17 | 25 | 23 | 23 | 36 | 30 | 0.23-0.27 | 7 | 40 |
| Pelvis | Pubis ramus superior (lower) | 19 | 20 | 20 |  |  | 30  12 | 0.12-0.23 | 10 | 12 |
| Pelvis | Pubis ramus superior (upper) | 8 | 20 | 20 |  |  | 30  12 | 0.12-0.23 | 10 | 12 |
| Ribs | 1, 2 | 12 |  | 14 |  |  | 38 | 0.05-0.25 | 12 | 11 |
| Ribs | 11, 12 | 18 |  | 17 |  |  | 38 | 0.05-0.25 | 12 | 11 |
| Ribs | 3, 4, 9, 10 | 8 |  | 14 |  |  | 38 | 0.05-0.25 | 12 | 11 |
| Ribs | 5,6,7,8 | 14 |  | 13 |  |  | 38 | 0.05-0.25 | 12 | 11 |
| Sacrum | Ala 3-4 | 16 | 9 | 15 | 15 |  | 8 | 0.1-0.19 | 13 | 16 |
| Sacrum | Body 1 | 7 | 11 | 10 |  |  | 8 | 0.1-0.19 | 13 | 16 |
| Sacrum | Body 2-3 | 8 | 11 | 9 |  |  | 8 | 0.1-0.19 | 13 | 16 |
| Sacrum | Body 4-5 | 9 | 11 | 13 |  |  | 8 | 0.1-0.19 | 13 | 16 |
| Sacrum | Pedicle 1 | 14 | 15 | 11 |  |  | 8 | 0.1-0.19 | 13 | 16 |
| Sacrum | Pedicle 2 | 14 | 11 | 17 |  |  | 8 | 0.1-0.19 | 13 | 16 |
| Sacrum | Pedicle 3 | 14 | 11 | 14 |  |  | 8 | 0.1-0.19 | 13 | 16 |
| Sacrum | Pedicle 4 | 14 | 11 | 18 |  |  | 8 | 0.1-0.19 | 13 | 16 |
| Sacrum | Sacral ala 1 | 13 | 10 | 13 |  |  | 8 | 0.1-0.19 | 13 | 16 |
| Sacrum | Sacral ala 2 | 15 | 17 | 8 |  |  | 8 | 0.1-0.19 | 13 | 16 |
| Scapula | Acromion | 18 | 11 | 11 |  |  | 13 | 0.09-0.47 | 42 | 23 |
| Scapula | Glenoid | 9 | 9 | 10 |  |  | 28 | 0.09-0.47 | 42 | 23 |
| Scapula | Lateral margin |  | 10 | 13 |  |  | 13 | 0.09-0.47 | 42 | 23 |
| Skull | Flat bones | 12 |  |  |  |  | 33  22 | 0.41-0.65 | 32 | 35 |
| Sternum | Sternum bdoy | 10 |  |  |  |  | 42 | 0.08-0.22 | 29 | 9 |
| Vertebra | C-body 3-7 | 16 | 14 | 12 |  |  | 7 | 0.16-0.28 | 14 | 10 |
| Vertebra | Cervical body 2 | 13 | 10 | 3 |  |  | 7 | 0.16-0.28 | 14 | 10 |
| Vertebra | Cervical lateral 1 | 13 | 9 | 9 |  |  | 7 | 0.16-0.28 | 14 | 10 |
| Vertebra | L- lamina+inf.pr | 10 | 13 | 17 |  |  | 34 | 0.1-0.19 | 13 | 16 |
| Vertebra | L-body | 12 | 8 | 9 |  |  | 16 | 0.11-0.28 | 15 | 15 |
| Vertebra | L-spinous pr. | 13 | 6 | 17 |  |  | 50 | 0.1-0.19 | 13 | 16 |
| Vertebra | L-superior pr. | 14 | 13 | 17 |  |  | 34 | 0.1-0.19 | 13 | 16 |
| Vertebra | L-transverse pr. | 8 | 9 | 13 |  |  | 50 | 0.1-0.19 | 13 | 16 |
| Vertebra | T- body | 7 | 9 | 11 |  |  | 6 | 0.16-0.28 | 14 | 10 |
| Vertebra | T- lamina+inf. | 12 | 14 | 13 |  |  | 16 | 0.11-0.28 | 15 | 15 |
| Vertebra | T-spinous pr. | 15 | 4 | 20 |  |  | 16 | 0.11-0.28 | 15 | 15 |
| Vertebra | T-superior pr. | 12 | 14 | 11 |  |  | 16 | 0.11-0.28 | 15 | 15 |
| Vertebra | T-transverse pr. | 9 | 11 | 13 |  |  | 16 | 0.11-0.28 | 15 | 15 |

Table S1.8 Coefficients of variation (%) of parameters of bone segment phantoms and a range of possible values is specified for adult female

| Site | Segment | h | a | b | c | d | Ct.Th | BV/TV | Tb.Th | Tb.Sp |
| --- | --- | --- | --- | --- | --- | --- | --- | --- | --- | --- |
| Clavicle | Acromial end |  | 16 | 14 |  |  | 19 | 0.15-0.46 | 13 | 25 |
| Clavicle | Shaft | 6 | 16 | 14 | 10 | 9 | 2 | 0.08-0.18 | 31 | 25 |
| Clavicle | Sternal end | 6 | 14 | 10 | 10 | 9 | 2 | 0.08-0.18 | 31 | 25 |
| Femur | Neck | 14 | 10 | 9 |  |  | 5 | 0.14-0.22 | 19 | 13 |
| Femur | Trochanter area | 5 | 7 | 7 | 6 | 6 | 15 | 0.08-0.13 | 65 | 20 |
| Humerus | Proximal end | 10 | 6 | 6 | 11 | 11 | 18 | 0.01-0.13 | 18 | 25 |
| Pelvis | Acetabulum | 10 | 10 | 20 |  |  | 30  30 | 0.11-0.25 | 15 | 20 |
| Pelvis | Iliac ala | 31 |  |  |  |  | 30 | 0.11-0.25 | 15 | 20 |
| Pelvis | Iliac crest | 15 |  | 9 |  |  | 30 | 0.11-0.25 | 15 | 20 |
| Pelvis | Iliac dorsal segment | 16 |  |  |  |  | 30 | 0.11-0.25 | 15 | 20 |
| Pelvis | Ischium ramus |  | 9 | 8 |  |  | 30 | 0.23-0.27 | 7 | 40 |
| Pelvis | Pubis ramus inferior | 17 | 25 | 23 | 23 | 36 | 30 | 0.23-0.27 | 7 | 40 |
| Pelvis | Pubis ramus superior (lower | 13 | 7 | 18 |  |  | 30  12 | 0.12-0.23 | 10 | 12 |
| Pelvis | Pubis ramus superior (upper | 7 | 18 | 20 |  |  | 30  12 | 0.12-0.23 | 10 | 12 |
| Ribs | 1, 2 | 11 |  | 15 |  |  | 38 | 0.05-0.25 | 12 | 11 |
| Ribs | 11, 12 | 11 |  | 25 |  |  | 38 | 0.05-0.25 | 12 | 11 |
| Ribs | 3, 4, 9, 10 | 11 |  | 14 |  |  | 38 | 0.05-0.25 | 12 | 11 |
| Ribs | 5.6.7.8 | 11 |  | 13 |  |  | 38 | 0.05-0.25 | 12 | 11 |
| Sacrum | Ala 3-4 | 16 | 9 | 15 | 15 |  | 8 | 0.1-0.19 | 13 | 16 |
| Sacrum | Body 1 | 9 | 11 | 12 |  |  | 8 | 0.1-0.19 | 13 | 16 |
| Sacrum | Body 2-3 | 15 | 11 | 13 |  |  | 8 | 0.1-0.19 | 13 | 16 |
| Sacrum | Body 4-5 | 14 | 11 | 12 |  |  | 8 | 0.1-0.19 | 13 | 16 |
| Sacrum | Pedicle 1 | 14 | 15 | 11 |  |  | 8 | 0.1-0.19 | 13 | 16 |
| Sacrum | Pedicle 2 | 14 | 11 | 17 |  |  | 8 | 0.1-0.19 | 13 | 16 |
| Sacrum | Pedicle 3 | 14 | 11 | 14 |  |  | 8 | 0.1-0.19 | 13 | 16 |
| Sacrum | Pedicle 4 | 14 | 11 | 18 |  |  | 8 | 0.1-0.19 | 13 | 16 |
| Sacrum | Sacral ala 1 | 9 | 15 | 8 |  |  | 8 | 0.1-0.19 | 13 | 16 |
| Sacrum | Sacral ala 2 | 9 | 17 | 13 |  |  | 8 | 0.1-0.19 | 13 | 16 |
| Scapula | Acromion | 18 | 11 | 11 |  |  | 13 | 0.09-0.47 | 42 | 23 |
| Scapula | Glenoid | 9 | 9 | 10 |  |  | 28 | 0.09-0.47 | 42 | 23 |
| Scapula | Lateral margin |  | 10 | 13 |  |  | 13 | 0.09-0.47 | 42 | 23 |
| Skull | Flat bones | 12 |  |  |  |  | 33  22 | 0.41-0.65 | 32 | 35 |
| Sternum | Manubrium | 15 |  |  |  |  | 42 | 0.08-0.22 | 29 | 9 |
| Vertebra | C- body 3-7 | 6 | 6 | 8 |  |  | 7 | 0.16-0.28 | 14 | 10 |
| Vertebra | Cervical body 2 | 13 | 10 | 3 |  |  | 7 | 0.16-0.28 | 14 | 10 |
| Vertebra | Cervical lateral 1 | 13 | 9 | 9 |  |  | 7 | 0.16-0.28 | 14 | 10 |
| Vertebra | L- body | 7 | 8 | 8 |  |  | 16 | 0.11-0.28 | 15 | 15 |
| Vertebra | L- lamina+inf.pr | 10 | 13 | 17 |  |  | 34 | 0.1-0.19 | 13 | 16 |
| Vertebra | L-spinous pr. | 15 | 16 | 17 |  |  | 50 | 0.1-0.19 | 13 | 16 |
| Vertebra | L-superior pr. | 14 | 13 | 17 |  |  | 34 | 0.1-0.19 | 13 | 16 |
| Vertebra | L-transverse pr. | 8 | 9 | 13 |  |  | 50 | 0.1-0.19 | 13 | 16 |
| Vertebra | T- body | 6 | 6 | 8 |  |  | 6 | 0.16-0.28 | 14 | 10 |
| Vertebra | T- lamina+inf. | 12 | 14 | 13 |  |  | 16 | 0.11-0.28 | 15 | 15 |
| Vertebra | T-spinous pr. | 15 | 4 | 20 |  |  | 16 | 0.11-0.28 | 15 | 15 |
| Vertebra | T-superior pr. | 12 | 14 | 11 |  |  | 16 | 0.11-0.28 | 15 | 15 |
| Vertebra | T-transverse pr. | 9 | 11 | 13 |  |  | 16 | 0.11-0.28 | 15 | 15 |
