# Supplementary material for: Stochastic parametric skeletal dosimetry model for humans: Pediatric and adult computational skeleton phantoms for internal bone marrow dosimetry
Source: PLoS One. 2025 Jul 3;20(7):e0327479. doi: 10.1371/journal.pone.0327479 (PMC12225816; doi:10.1371/journal.pone.0327479)
Supplement: S2 File — (DOCX) [file pone.0327479.s002.docx]

**Supplementary material S2. Segment-specific volumes of trabecular bone, cortical bone, and bone marrow**

In tables S2.1-S2.8 segment-specific volumes of trabecular bone (TBV), cortical bone (CBV), and bone marrow (BMV) for different ages are presented. The range of medium volumes depends on individual variability of linear dimensions and corresponds to 90% confidence interval (CI). *n_s_*- the number of similar models; *k_s_*-linear or area proportion factor, used for recalculation of truncated segment volume to whole segment volume.

Table S2.1 Segment-specific volumes for newborns.

| Site | Segment | *n_s_×k_s_* | Voxel resolution | volume, cm^3^ | | | | | |
| --- | --- | --- | --- | --- | --- | --- | --- | --- | --- |
|  |  |  |  | TBV | | BMV | | CBV | |
|  |  |  |  | reference | 90% CI | reference | 90% CI | reference | 90% CI |
| Clavicle | Acromeon end | 2 | 100 | 0.08 | 0.02-0.2 | 0.22 | 0.06-0.6 | 0.08 | 0.02-0.16 |
| Clavicle | Shaft | 2 | 100 | 0.18 | 0.02-0.34 | 0.44 | 0.04-1.02 | 0.72 | 0.38-1.52 |
| Clavicle | Sternal end | 2 | 100 | 0.16 | 0.06-0.32 | 0.38 | 0.1-0.84 | 0.10 | 0.04-0.14 |
| Femur | Distal end | 2 | 70 | 1.58 | 1.04-1.94 | 2.72 | 1.98-3.06 | 0.60 | 0.38-0.86 |
| Femur | Proximal end | 2 | 70 | 1.52 | 0.94-1.86 | 2.64 | 2.4-4.3 | 0.74 | 0.6-1.08 |
| Femur | Shaft | 2×1.261 | 70 | 0.33 | 0.18-0.6 | 0.55 | 0.3-1.31 | 2.22 | 1.84-3.15 |
| Hand and foot | Precarpal | 4 | 80 | 0.28 | 0.08-0.68 | 1.04 | 0.64-1.92 | 0.24 | 0.16-0.44 |
| Hand and foot | Tube bones | 50 | 80 | 1.00 | 0-1.5 | 3.00 | 0-4 | 1.00 | 0.5-1.5 |
| Humerus | Distal end | 2 | 70 | 0.34 | 0.16-0.38 | 0.88 | 0.54-1.7 | 0.22 | 0.14-0.26 |
| Humerus | Proximal end | 2 | 70 | 0.48 | 0.16-0.86 | 1.22 | 0.72-1.64 | 0.30 | 0.22-0.44 |
| Humerus | Shaft | 2×1.341 | 70 | 0.21 | 0.05-0.32 | 0.54 | 0.19-0.91 | 1.55 | 1.07-2.39 |
| Pelvis | Ilium part 1 | 2 | 110 | 0.84 | 0.14-1.62 | 1.78 | 0.7-3.84 | 1.88 | 1.36-2.18 |
| Pelvis | Ilium part 2 | 2 | 110 | 0.84 | 0.26-1.24 | 1.84 | 1.2-2.8 | 0.34 | 0.32-0.42 |
| Pelvis | Ischium | 2 | 110 | 0.74 | 0.18-0.98 | 1.60 | 0.86-2.82 | 0.22 | 0.16-0.36 |
| Pelvis | Pubis | 2 | 110 | 0.36 | 0.24-0.86 | 0.78 | 0.52-1.62 | 0.26 | 0.18-0.4 |
| Radius and ulna | End | 4 | 50 | 0.12 | 0.08-0.24 | 0.52 | 0.36-0.76 | 0.20 | 0.12-0.2 |
| Radius and ulna | Shaft | 2×3.34 | 50 | 0.13 | 0.07-0.2 | 0.54 | 0.33-1 | 1.74 | 1.2-2.01 |
| Ribs | Ribs | 2×38.042 | 100 | 5.33 | 1.52-9.13 | 22.06 | 9.89-50.21 | 14.46 | 6.85-22.06 |
| Sacrum | Body 1 | 1 | 70 | 0.31 | 0.11-0.36 | 0.39 | 0.2-0.66 | 0.00 | 0-0 |
| Sacrum | Body 2 | 1 | 70 | 0.20 | 0.06-0.31 | 0.25 | 0.18-0.53 | 0.00 | 0-0 |
| Sacrum | Body 3 | 1 | 70 | 0.12 | 0.04-0.17 | 0.15 | 0.09-0.32 | 0.00 | 0-0 |
| Sacrum | Body 4 | 1 | 70 | 0.08 | 0.03-0.12 | 0.10 | 0.06-0.17 | 0.00 | 0-0 |
| Sacrum | Body 5 | 1 | 70 | 0.05 | 0.02-0.08 | 0.06 | 0.03-0.12 | 0.00 | 0-0 |
| Scapula | Acromion | 2 | 90 | 0.68 | 0.16-1.1 | 1.70 | 0.78-4.22 | 0.54 | 0.3-0.8 |
| Scapula | Body | 2×0.793 | 90 | 0.79 | 0.4-1.07 | 2.02 | 1.6-2.45 | 1.03 | 1.03-1.28 |
| Scapula | Glenoid | 2 | 90 | 0.14 | 0.04-0.32 | 0.38 | 0.16-0.58 | 0.12 | 0.08-0.16 |
| Skull | Flat bones | 18.23 | 200 | 17.29 | 12.19-23.84 | 15.47 | 11.65-22.57 | 0.00 | 0-0 |
| Tibia and fibula | Distal end | 2 | 50 | 0.88 | 0.3-1.2 | 1.66 | 0.74-3.46 | 0.30 | 0.2-0.46 |
| Tibia and fibula | Fibula shaft | 2×2.231 | 50 | 0.09 | 0.04-0.13 | 0.18 | 0.04-0.31 | 0.62 | 0.45-0.89 |
| Tibia and fibula | Proximal end | 2 | 50 | 1.38 | 0.62-1.96 | 2.56 | 1.26-4.28 | 0.42 | 0.28-0.66 |
| Tibia and fibula | Tibia shaft | 2×1.361 | 50 | 0.38 | 0.08-0.87 | 0.71 | 0.14-1.39 | 1.99 | 1.06-2.67 |
| Vertebra | C-body | 7 | 200 | 0.56 | 0.35-0.7 | 0.42 | 0.35-0.77 | 0.00 | 0-0 |
| Vertebra | L-body | 5 | 70 | 1.45 | 0.7-1.85 | 1.80 | 1.15-2.7 | 0.00 | 0-0 |
| Vertebra | T-body | 12 | 70 | 1.80 | 0.72-2.04 | 2.16 | 1.08-3.72 | 0.00 | 0-0 |

Table S2.2 Segment-specific volumes for 1- year-old child

| Site | Segment | *n_s_×k_s_* | Voxel resolution | volume, cm^3^ | | | | | |
| --- | --- | --- | --- | --- | --- | --- | --- | --- | --- |
|  |  |  |  | TBV | | BM | | CBV | |
|  |  |  |  | reference | 90% CI | reference | 90% CI | reference | 90% CI |
| Clavicle | Acromial end | 2 | 100 | 0.16 | 0.04-0.34 | 0.40 | 0.24-0.64 | 0.14 | 0.1-0.2 |
| Clavicle | Shaft | 2 | 100 | 0.36 | 0.14-0.8 | 0.86 | 0.5-1.68 | 1.26 | 0.8-1.8 |
| Clavicle | Sternal end | 2 | 100 | 0.28 | 0.12-0.48 | 0.70 | 0.48-1.12 | 0.18 | 0.12-0.26 |
| Femur | Distal end | 2 | 110 | 3.76 | 2.32-6.14 | 13.06 | 9.8-19.6 | 2.42 | 1.74-3.48 |
| Femur | Proximal end | 2 | 110 | 3.44 | 1.22-5.82 | 12.98 | 8.9-21.64 | 2.82 | 1.66-4.04 |
| Femur | Shaft | 2×2.381 | 110 | 1.09 | 0.43-2.24 | 3.81 | 2.14-5.71 | 9.19 | 6.85-11.61 |
| Humerus | Distal end | 2 | 130 | 0.62 | 0.16-1.8 | 2.24 | 1.06-4.58 | 0.46 | 0.2-0.78 |
| Humerus | Proximal end | 2 | 130 | 1.06 | 0.56-1.54 | 3.72 | 1.96-5.62 | 0.70 | 0.38-1.1 |
| Humerus | Shaft | 2×1.61 | 130 | 0.58 | 0.16-0.93 | 2.05 | 1.25-3.81 | 3.62 | 2.69-5.66 |
| Pelvis | Ilium acetabular part | 2 | 100 | 1.62 | 0.66-3.08 | 5.52 | 2.44-9.46 | 0.70 | 0.34-0.92 |
| Pelvis | Ilium flat part 1 | 2×1.283 | 100 | 1.74 | 0.84-2.05 | 5.86 | 3.99-8.42 | 3.92 | 3.23-4.38 |
| Pelvis | Ilium flat part 2 | 2×1.283 | 100 | 2.18 | 1.36-2.69 | 7.50 | 5.84-10.29 | 1.84 | 1.38-2.3 |
| Pelvis | Ischium acetabular part | 2 | 100 | 0.90 | 0.26-1.7 | 3.08 | 1.18-6.44 | 0.72 | 0.4-1.12 |
| Pelvis | Ischium tuberosity | 2 | 100 | 0.72 | 0.54-1.14 | 2.42 | 1.72-3.94 | 0.48 | 0.34-0.56 |
| Pelvis | Pubic ramus superior | 2 | 100 | 0.32 | 0.22-0.54 | 1.10 | 0.84-1.72 | 0.36 | 0.24-0.68 |
| Pelvis | Pubis acetabular part | 2 | 100 | 0.16 | 0.06-0.68 | 0.54 | 0.18-1.88 | 0.12 | 0.06-0.2 |
| Radius and ulna | End | 4 | 90 | 0.24 | 0.08-0.36 | 1.12 | 0.92-1.6 | 0.44 | 0.28-0.6 |
| Radius and ulna | Shaft | 2×4.75 | 90 | 0.29 | 0.1-0.76 | 1.71 | 1.05-2.85 | 4.18 | 3.42-4.85 |
| Ribs | Ribs | 2×45.242 | 150 | 17.19 | 3.62-46.14 | 42.53 | 16.29-126.67 | 32.57 | 22.62-52.48 |
| Sacrum | Ala 1 | 2 | 70 | 0.32 | 0.14-0.6 | 2.14 | 1.38-3.56 | 0.00 | 0-0 |
| Sacrum | Ala 2 | 2 | 70 | 0.20 | 0.1-0.38 | 1.26 | 0.86-2.1 | 0.00 | 0-0 |
| Sacrum | Ala 3 | 2 | 70 | 0.16 | 0.08-0.3 | 1.02 | 0.5-1.32 | 0.00 | 0-0 |
| Sacrum | Ala 4 | 2 | 70 | 0.08 | 0.04-0.1 | 0.46 | 0.24-0.64 | 0.00 | 0-0 |
| Sacrum | Body 1 | 1 | 70 | 0.40 | 0.14-1.01 | 2.50 | 1.32-4.19 | 0.00 | 0-0 |
| Sacrum | Body 2 | 1 | 70 | 0.25 | 0.1-0.51 | 1.60 | 0.99-2.96 | 0.00 | 0-0 |
| Sacrum | Body 3 | 1 | 70 | 0.15 | 0.07-0.25 | 0.97 | 0.59-1.73 | 0.00 | 0-0 |
| Sacrum | Body 4 | 1 | 70 | 0.10 | 0.03-0.14 | 0.64 | 0.34-0.92 | 0.00 | 0-0 |
| Sacrum | Body 5 | 1 | 70 | 0.05 | 0.03-0.08 | 0.30 | 0.22-0.46 | 0.00 | 0-0 |
| Scapula | Acromion | 2 | 130 | 0.52 | 0.1-3.24 | 1.86 | 0.28-5.54 | 0.54 | 0.18-1.32 |
| Scapula | Body | 2×0.953 | 130 | 0.72 | 0.34-0.89 | 2.62 | 1.92-3.74 | 1.33 | 1.33-1.56 |
| Scapula | Glenoid | 2 | 130 | 0.36 | 0.04-0.86 | 1.28 | 0.28-1.5 | 0.26 | 0.1-0.26 |
| Skull | Flat bones | 30.93 | 200 | 20.10 | 8.97-39.59 | 18.87 | 7.73-38.35 | 38.97 | 33.4-50.11 |
| Tibia and fibula | Distal end | 2 | 70 | 1.04 | 0.42-2.48 | 4.08 | 1.72-7.12 | 0.88 | 0.5-1.3 |
| Tibia and fibula | Fibula shaft | 2×3.51 | 70 | 0.14 | 0-0.35 | 0.56 | 0.14-0.91 | 2.52 | 1.89-3.57 |
| Tibia and fibula | Proximal end | 2 | 70 | 2.46 | 1.34-6.3 | 9.68 | 5.54-13.76 | 1.82 | 1.26-2.48 |
| Tibia and fibula | Tibia shaft | 2×1.671 | 70 | 0.30 | 0.13-0.63 | 1.20 | 0.53-2.07 | 4.84 | 2.94-6.68 |
| Vertebra | Cervical vertebra body | 7 | 130 | 0.77 | 0.42-1.19 | 3.15 | 2.17-3.64 | 0.00 | 0-0 |
| Vertebra | Lumbar vertebra body | 5 | 70 | 1.00 | 0.65-2.05 | 6.60 | 4.9-9.3 | 0.00 | 0-0 |
| Vertebra | Thoracic vertebra body | 12 | 70 | 1.80 | 0.72-4.68 | 11.64 | 5.52-17.76 | 0.00 | 0-0 |

Table S2.3 Segment-specific volumes for 5- year-old child

| Site | Segment | *n_s_×k_s_* | Voxel resolution | volume, cm^3^ | | | | | |
| --- | --- | --- | --- | --- | --- | --- | --- | --- | --- |
|  |  |  |  | TBV | | BM | | CBV | |
|  |  |  |  | reference | 90% CI | reference | 90% CI | reference | 90% CI |
| Clavicle | Acromial end | 2 | 100 | 0.42 | 0.16-0.88 | 1.00 | 0.4-2.06 | 0.34 | 0.26-0.5 |
| Clavicle | Shaft | 2×2.31 | 130 | 0.46 | 0.18-0.87 | 2.76 | 1.2-3.54 | 3.17 | 2.07-3.82 |
| Clavicle | Sternal end | 2 | 100 | 0.72 | 0.3-1.24 | 1.78 | 0.92-3.16 | 0.44 | 0.28-0.64 |
| Femur | Distal end | 2 | 160 | 15.12 | 8.62-21.24 | 45.80 | 33.78-58 | 10.42 | 8.68-11.96 |
| Femur | Lower proximal end | 2 | 160 | 5.68 | 1.4-13.76 | 10.48 | 3.94-19.06 | 4.38 | 2.4-6.66 |
| Femur | Shaft | 2×4.931 | 160 | 5.13 | 2.17-8.48 | 14.59 | 10.45-20.31 | 44.57 | 41.81-53.54 |
| Femur | Upper proximal end | 2 | 160 | 5.64 | 3.44-7.42 | 10.58 | 8.6-13.92 | 4.24 | 3.7-4.76 |
| Humerus | Distal end | 2 | 150 | 3.44 | 1.96-4.98 | 12.68 | 9.92-15.84 | 2.78 | 1.96-3.7 |
| Humerus | Proximal end | 2 | 150 | 3.30 | 1.48-5.84 | 12.70 | 8.92-14.6 | 2.62 | 2.08-2.78 |
| Humerus | Shaft | 2×2.941 | 150 | 2.82 | 0.76-3.7 | 10.17 | 8.17-15.58 | 16.99 | 14.52-19.87 |
| Pelvis | Ilium acetabular part | 2 | 110 | 5.12 | 2.14-10.78 | 15.24 | 6.54-33.78 | 2.80 | 1.76-4.32 |
| Pelvis | Ilium flat part 1 | 2×2.353 | 110 | 5.69 | 4.32-7.38 | 17.63 | 14.95-22.47 | 10.25 | 9.78-11.19 |
| Pelvis | Ilium flat part 2 | 2×2.353 | 110 | 6.53 | 4.47-9.21 | 20.07 | 16.31-24.35 | 7.00 | 6.06-7.94 |
| Pelvis | Ischial ramus inferior | 2 | 110 | 0.44 | 0.18-1 | 1.34 | 0.62-3.42 | 0.50 | 0.22-1.14 |
| Pelvis | Ischium acetabular part | 2 | 110 | 1.96 | 1-3 | 5.88 | 3.44-12.64 | 1.46 | 1-2.26 |
| Pelvis | Ischium tuberosity | 2 | 110 | 2.80 | 1.54-3.64 | 8.38 | 4.7-12.68 | 1.54 | 0.88-1.96 |
| Pelvis | Pubis acetabular part | 2 | 110 | 0.60 | 0.24-1 | 1.78 | 0.8-2.7 | 0.34 | 0.2-0.52 |
| Pelvis | Pubis ramus inferior | 2 | 110 | 0.44 | 0.18-1 | 1.34 | 0.62-3.42 | 0.50 | 0.22-1.14 |
| Pelvis | Pubis ramus superior | 2 | 110 | 1.06 | 0.6-2 | 3.22 | 1.58-6.6 | 0.96 | 0.7-1.2 |
| Radius and ulna | End | 4 | 130 | 0.92 | 0.56-1.6 | 4.72 | 3.76-6 | 1.44 | 1.12-2.12 |
| Radius and ulna | Shaft | 4×3.82 | 130 | 1.68 | 0.76-3.66 | 8.86 | 6.41-13.13 | 14.35 | 11.76-17.71 |
| Ribs | Ribs | 2×64.652 | 180 | 20.69 | 6.47-40.08 | 84.05 | 19.4-197.83 | 53.01 | 18.1-87.92 |
| Sacrum | Body-ala 1 | 1 | 70 | 3.46 | 1.72-5.29 | 22.21 | 12.79-33.95 | 1.62 | 0.91-2.78 |
| Sacrum | Body-ala 2 | 1 | 70 | 1.71 | 0.84-2.81 | 10.86 | 6.79-22.62 | 1.16 | 0.74-1.71 |
| Sacrum | Body-ala 3 | 1 | 70 | 0.91 | 0.36-1.3 | 5.87 | 3.49-11.01 | 0.90 | 0.58-1.38 |
| Sacrum | Body-ala 4 | 1 | 70 | 0.32 | 0.14-0.51 | 2.03 | 0.89-3.08 | 0.58 | 0.24-0.86 |
| Sacrum | Body-ala 5 | 1 | 70 | 0.16 | 0.06-0.27 | 1.02 | 0.5-1.64 | 0.29 | 0.15-0.44 |
| Scapula | Acromion | 2 | 180 | 0.72 | 0.28-1.68 | 2.66 | 1-5.2 | 1.38 | 0.88-2.12 |
| Scapula | Glenoid | 2 | 180 | 1.48 | 0.56-2.84 | 5.66 | 3.88-9.24 | 1.40 | 0.86-2.04 |
| Scapula | Lateral margin | 2×2.451 | 180 | 0.49 | 0.15-0.74 | 1.76 | 1.23-3.19 | 2.60 | 2.06-3.48 |
| Skull | Flat bones | 1×35.353 | 200 | 36.41 | 5.66-48.43 | 33.58 | 7.07-53.38 | 63.63 | 50.9-82.72 |
| Sternum | Sternum | 1.8 | 90 | 1.60 | 0.92-1.89 | 9.02 | 8.19-10.46 | 0.58 | 0.14-0.72 |
| Tibia and fibula | Distal end | 2 | 90 | 4.56 | 1.54-9.72 | 13.58 | 7.56-26.02 | 2.64 | 1.9-3.16 |
| Tibia and fibula | Fibula body | 2×6.621 | 90 | 1.99 | 1.06-3.05 | 6.09 | 4.77-9 | 12.45 | 8.61-14.43 |
| Tibia and fibula | Proximal end | 2 | 90 | 7.90 | 4.84-9.94 | 25.20 | 17.9-30.16 | 3.74 | 2.34-4.54 |
| Tibia and fibula | Tibia shaft | 2×4.561 | 90 | 4.92 | 1.92-5.29 | 14.87 | 9.48-16.78 | 31.10 | 24.35-36.12 |
| Vertebra | C-body | 7 | 100 | 1.68 | 0.77-2.1 | 6.23 | 4.27-9.31 | 0.35 | 0.28-0.42 |
| Vertebra | L-body | 2 | 80 | 6.70 | 2.9-12.15 | 42.55 | 29.5-69.45 | 1.50 | 0.95-2.25 |
| Vertebra | L-spinous proc | 2 | 70 | 0.60 | 0.35-1.05 | 3.65 | 2.15-6.15 | 0.55 | 0.35-0.75 |
| Vertebra | L-transverse proc | 2 | 70 | 0.50 | 0.2-0.7 | 2.90 | 1.9-5.1 | 0.50 | 0.5-0.8 |
| Vertebra | T-body | 12 | 70 | 5.28 | 1.32-13.32 | 34.32 | 14.64-84.72 | 1.68 | 0.96-2.28 |
| Vertebra | T-spinous proc | 2×4.931 | 70 | 0.15 | 0.1-0.2 | 1.05 | 0.35-1.9 | 0.30 | 0.15-0.45 |
| Vertebra | T-transverse proc | 5 | 70 | 1.20 | 0.24-1.68 | 7.44 | 3.36-8.64 | 1.44 | 0.72-1.68 |

Table S2.4 Segment-specific volumes for 10- year-old child

| Site | Segment | *n_s_×k_s_* | Voxel resolution | volume, cm^3^ | | | | | |
| --- | --- | --- | --- | --- | --- | --- | --- | --- | --- |
|  |  |  |  | TBV | | BM | | CBV | |
|  |  |  |  | reference | 90% CI | reference | 90% CI | reference | 90% CI |
| Clavicle | Acromial end | 2 | 100 | 0.82 | 0.38-1.44 | 2.02 | 1.28-2.68 | 0.92 | 0.58-1.26 |
| Clavicle | Body | 2×3.15 | 130 | 0.82 | 0.32-2.33 | 4.35 | 2.52-8.38 | 8.32 | 5.23-11.03 |
| Clavicle | Sternal end | 2 | 100 | 1.46 | 0.52-1.62 | 3.60 | 2.1-5.24 | 1.16 | 0.84-1.5 |
| Femur | Distal end | 2 | 160 | 34.98 | 17.74-47.86 | 99.88 | 78.82-138.12 | 17.94 | 13.26-22.82 |
| Femur | Lower proximal end | 2.00×1.15 | 160 | 7.96 | 4.72-9.61 | 14.74 | 10.49-20.93 | 10.99 | 8.76-12.1 |
| Femur | Upper proximal end | 2.00×1.15 | 160 | 8.67 | 5.06-10.03 | 15.99 | 14.44-23.02 | 9.04 | 7.48-11.57 |
| Humery | Distal end | 2 | 140 | 5.20 | 3.16-8.04 | 18.54 | 13.34-26.12 | 3.48 | 2.76-3.88 |
| Humery | Proximal end | 2 | 140 | 5.70 | 2.9-8.28 | 21.50 | 15.4-25.24 | 4.86 | 3.8-5.58 |
| Pelvis | Ilium acetabular part | 2 | 110 | 8.58 | 3.84-31.96 | 28.32 | 13.6-95.76 | 2.06 | 1-3.82 |
| Pelvis | Ilium flat part 1 | 2×4.34 | 110 | 10.25 | 4.78-13.38 | 31.96 | 17.72-46.12 | 20.67 | 18.94-20.67 |
| Pelvis | Ilium flat part 2 | 2×4.34 | 110 | 11.99 | 7.99-11.64 | 37.18 | 26.49-39.09 | 13.81 | 12.07-14.68 |
| Pelvis | Ischial ramus inferior | 2 | 110 | 1.10 | 0.58-1.78 | 3.36 | 2.02-6.26 | 1.00 | 0.46-1.8 |
| Pelvis | Ischium acetabular part | 2 | 110 | 3.02 | 1.36-6.58 | 9.16 | 4.98-19.46 | 2.16 | 1.54-3.38 |
| Pelvis | Ischium tuberosity | 2 | 110 | 5.38 | 3.82-10.14 | 16.42 | 12.06-30.5 | 2.62 | 1.78-3.1 |
| Pelvis | Pubis acetabular part | 2 | 110 | 1.22 | 0.6-1.66 | 3.70 | 2.44-6.48 | 0.56 | 0.28-0.9 |
| Pelvis | Pubis ramus inferior | 2 | 110 | 1.10 | 0.58-1.78 | 3.36 | 2.02-6.26 | 1.00 | 0.46-1.8 |
| Pelvis | Pubis ramus superior | 2 | 110 | 2.14 | 1.34-3.22 | 6.52 | 4.4-11.24 | 1.58 | 0.88-2.1 |
| Ribs | Ribs | 2×79.4 | 180 | 28.58 | 19.06-46.05 | 115.92 | 47.64-187.38 | 103.22 | 66.7-117.51 |
| Sacrum | Body-ala 1 | 1 | 80 | 6.12 | 1.89-10.03 | 38.97 | 17.87-64.23 | 4.50 | 2.54-7.14 |
| Sacrum | Body-ala 2 | 1 | 80 | 2.46 | 0.77-5.24 | 15.88 | 9.97-59.93 | 3.00 | 2.07-6.44 |
| Sacrum | Body-ala 3 | 1 | 85 | 1.24 | 0.47-2.91 | 7.80 | 3.93-18.9 | 2.03 | 0.94-4.57 |
| Sacrum | Body-ala 4 | 1 | 85 | 0.55 | 0.2-0.79 | 3.53 | 1.84-6.1 | 1.30 | 0.58-2.07 |
| Sacrum | Body-ala 5 | 1 | 85 | 0.47 | 0.15-0.96 | 2.93 | 1.18-8.27 | 1.08 | 0.47-2.65 |
| Scapula | Acromion | 2 | 180 | 1.44 | 0.56-2.12 | 5.32 | 2.48-6.96 | 2.52 | 1.8-2.86 |
| Scapula | Glenoid | 2 | 180 | 2.32 | 1.02-3.46 | 8.34 | 5.24-10.68 | 1.88 | 1.3-2.22 |
| Scapula | Lateral margin | 2×3.12 | 180 | 0.75 | 0.37-1.19 | 2.68 | 1.25-3.18 | 3.00 | 2.31-3.56 |
| Skull | Flat bones | 1×35.7 | 200 | 36.77 | 17.49-63.9 | 33.92 | 21.06-58.19 | 77.11 | 57.83-77.11 |
| Sternum | Sternum | 4.40 | 90 | 4.27 | 2.82-6.56 | 24.20 | 20.28-30.36 | 4.97 | 3.56-5.32 |
| Tibia and fibula | Distal end | 2 | 140 | 11.92 | 6.3-15.96 | 40.80 | 28.88-44.52 | 5.74 | 4.32-6.28 |
| Tibia and fibula | Fibula ends | 4×1.58 | 150 | 2.21 | 1.58-2.47 | 6.83 | 5.06-10.24 | 9.68 | 7.02-10.5 |
| Tibia and fibula | Proximal end | 2 | 140 | 20.50 | 10.66-17.78 | 58.92 | 38.7-71.2 | 7.26 | 5.34-9.02 |
| Vertebra | C-body | 7 | 100 | 2.66 | 1.12-3.85 | 10.01 | 6.79-14.91 | 0.49 | 0.42-0.56 |
| Vertebra | L-body | 5 | 70 | 9.85 | 2.2-21.45 | 62.10 | 24.8-113 | 1.90 | 1.05-2.9 |
| Vertebra | L-spinous proc | 5 | 70 | 1.50 | 0.45-3.35 | 9.15 | 4.45-23.65 | 1.35 | 0.75-2.45 |
| Vertebra | L-transverse proc | 10 | 70 | 0.90 | 0.3-1.3 | 5.40 | 2-14.1 | 1.00 | 0.4-1.4 |
| Vertebra | T-body | 12 | 70 | 10.44 | 3.24-18.6 | 67.68 | 19.32-103.2 | 2.64 | 1.08-3 |
| Vertebra | T-spinous proc | 12 | 70 | 1.08 | 0.24-3.6 | 6.36 | 1.8-14.88 | 1.44 | 0.84-2.4 |
| Vertebra | T-transverse proc | 24 | 70 | 2.40 | 0.72-4.56 | 14.64 | 6-24.72 | 2.40 | 1.44-3.6 |

Table S2.5 Segment-specific volumes for 15- year-old male

| Site | Segment | *n_s_×k_s_* | Voxel resolution | volume, cm^3^ | | | | | |
| --- | --- | --- | --- | --- | --- | --- | --- | --- | --- |
|  |  |  |  | TBV | | BM | | CBV | |
|  |  |  |  | reference | 90% CI | reference | 90% CI | reference | 90% CI |
| Clavicle | Acromial end | 2 | 130 | 1.46 | 0.72-3.28 | 3.56 | 2.06-6.08 | 1.32 | 0.94-1.7 |
| Clavicle | Shaft | 2×3.94 | 100 | 1.97 | 1.02-3.31 | 11.11 | 4.57-15.84 | 13.71 | 8.35-17.73 |
| Clavicle | Sternal end | 2 | 100 | 2.64 | 1.32-5.76 | 6.74 | 4.52-9.44 | 1.70 | 1.14-2.84 |
| Femur | Neck | 2 | 140 | 14.76 | 8.32-23.96 | 27.20 | 18.24-50.1 | 11.60 | 8.74-14.58 |
| Femur | Trochanter area | 2 | 150 | 22.86 | 16.2-30.96 | 70.24 | 50.8-80.54 | 23.78 | 20.32-26.42 |
| Humeri | Proximal end | 2 | 140 | 12.54 | 8.92-16.46 | 47.80 | 40.16-51.62 | 7.08 | 5.68-6.66 |
| Pelvis | Acetabulum | 2 | 110 | 7.50 | 0.34-18.72 | 22.50 | 0.88-55.32 | 12.90 | 6.02-21 |
| Pelvis | Iliac ala | 2×2.39 | 110 | 8.60 | 5.83-14.53 | 26.72 | 20.6-36.61 | 5.45 | 4.88-6.69 |
| Pelvis | Iliac crest | 2×5.37 | 100 | 8.81 | 4.94-11.38 | 26.64 | 18.69-41.78 | 10.63 | 7.95-13.32 |
| Pelvis | Iliac dorsal segment | 2×3.73 | 110 | 28.86 | 22.35-34.93 | 87.54 | 74.37-117.36 | 10.58 | 9.18-13.1 |
| Pelvis | Ischium ramus | 2×1.83 | 110 | 16.29 | 11.35-17.86 | 50.80 | 34.22-66.61 | 6.26 | 4.03-9.15 |
| Pelvis | Pubis ramus inferior | 2 | 110 | 5.98 | 2.04-12.32 | 18.62 | 7.52-52.04 | 2.80 | 1.3-4.96 |
| Pelvis | Pubis ramus superior (low) | 2 | 110 | 5.78 | 2.44-11.32 | 17.84 | 7.94-32.74 | 4.20 | 2.18-6.62 |
| Pelvis | Pubis ramus superior (upper part) | 2 | 110 | 4.92 | 2.06-6.58 | 15.08 | 6.38-18.76 | 3.68 | 2.24-4.22 |
| Ribs | 1, 2 | 2×12.43 | 60 | 7.71 | 3.73-14.17 | 56.43 | 38.28-90.74 | 24.86 | 18.65-27.1 |
| Ribs | 11, 12 | 2×42.53 | 60 | 12.76 | 6.8-24.67 | 97.82 | 58.69-150.56 | 56.99 | 42.53-73.15 |
| Ribs | 3, 4, 9, 10 | 2×38.62 | 60 | 17.76 | 6.95-23.93 | 129.70 | 77.97-136.64 | 64.08 | 45.55-74.11 |
| Ribs | 5,6,7,8 | 2×12.32 | 60 | 7.13 | 2.46-11.07 | 53.38 | 32.96-83.39 | 21.89 | 15.74-24.35 |
| Sacrum | Body 1 | 1 | 75 | 3.55 | 1.51-8.38 | 23.07 | 13.03-38.4 | 2.79 | 1.93-4.08 |
| Sacrum | Body 2-3 | 1 | 75 | 2.23 | 1.79-4.3 | 14.78 | 10.83-23.12 | 3.00 | 2.13-3.86 |
| Sacrum | Body 4-5 | 1 | 75 | 0.78 | 0.31-1.8 | 4.96 | 2.93-8.66 | 2.34 | 1.59-3.64 |
| Sacrum | Pedicle 1 | 2 | 75 | 0.80 | 0.52-1.74 | 5.22 | 2.8-8.94 | 1.84 | 1.02-2.36 |
| Sacrum | Pedicle 2 | 2 | 75 | 0.72 | 0.32-1.32 | 4.68 | 1.8-8.74 | 1.82 | 0.72-2.54 |
| Sacrum | Pedicle 3 | 2 | 75 | 0.50 | 0.18-0.82 | 3.16 | 1.42-4.74 | 1.44 | 0.64-1.46 |
| Sacrum | Pedicle 4 | 2 | 75 | 0.30 | 0.08-0.78 | 1.92 | 0.78-3.82 | 1.14 | 0.62-2.18 |
| Sacrum | Sacral ala 1 | 2 | 75 | 5.90 | 1.96-9.34 | 37.50 | 23.48-53.38 | 7.08 | 4.7-9.72 |
| Sacrum | Sacral ala 2 | 2 | 75 | 3.50 | 1-6.5 | 22.40 | 12.62-38.04 | 4.02 | 2.22-6.5 |
| Sacrum | Sacral ala 3-4 | 2 | 75 | 1.22 | 0.36-2.24 | 7.86 | 3.58-14.4 | 3.72 | 2.02-5.16 |
| Scapula | Acromion | 2 | 180 | 2.28 | 0.94-5.7 | 8.56 | 3.96-14.46 | 3.56 | 3.04-4.38 |
| Scapula | Glenoid | 2 | 180 | 3.36 | 2.26-7.82 | 12.38 | 7.64-20.66 | 2.46 | 1.8-3.58 |
| Scapula | Lateral margin | 2×3.52 | 180 | 0.84 | 0.35-1.69 | 3.03 | 1.9-3.8 | 3.38 | 2.46-4.44 |
| Skull | Flat bones | 1×37.723 | 200 | 46.02 | 31.31-66.76 | 42.25 | 29.8-55.45 | 88.26 | 81.48-101.84 |
| Sternum | Sternum | 1×7.08 | 90 | 8.22 | 4.11-13.03 | 47.88 | 34.14-60.63 | 11.47 | 8.57-15.44 |
| Vertebra | C1 mass | 2 | 60 | 0.72 | 0.28-1.26 | 2.68 | 1.44-4.94 | 2.54 | 1.84-3.72 |
| Vertebra | C2-body | 1 | 60 | 0.96 | 0.61-1.3 | 3.64 | 2.76-5.65 | 0.20 | 0.17-0.26 |
| Vertebra | C3-7 body | 5 | 100 | 2.60 | 0.8-3.45 | 9.95 | 6.75-13.4 | 0.95 | 0.7-1.2 |
| Vertebra | L- body | 5 | 70 | 17.50 | 10.65-23.5 | 112.65 | 68-139.4 | 5.85 | 3.05-6 |
| Vertebra | L-lamina+inf.pr. | 10 | 75 | 0.70 | 0.2-1.8 | 4.30 | 1.5-11 | 5.70 | 2.5-13.2 |
| Vertebra | L-spinous pr. | 5 | 70 | 2.35 | 0.8-3 | 14.50 | 9.3-21 | 4.00 | 2.85-4.4 |
| Vertebra | L-superior pr. | 10 | 50 | 2.90 | 1.2-3.5 | 18.70 | 10.1-21.4 | 3.60 | 1.7-4.4 |
| Vertebra | L-transverse pr. | 10 | 70 | 1.30 | 0.6-2.5 | 8.10 | 5.2-12.2 | 2.90 | 2.2-3.8 |
| Vertebra | T- body | 12 | 70 | 16.56 | 6.36-27.96 | 101.40 | 59.64-192.84 | 7.44 | 3.84-10.32 |
| Vertebra | T- lamina+inf. pr. | 24 | 60 | 1.68 | 0.24-3.84 | 11.04 | 1.2-29.04 | 20.16 | 11.04-29.28 |
| Vertebra | T-spinous pr. | 12 | 70 | 1.80 | 0.6-2.16 | 11.40 | 4.68-19.32 | 4.56 | 2.52-7.08 |
| Vertebra | T-superior pr. | 24 | 60 | 0.48 | 0.24-1.2 | 3.36 | 1.2-6.24 | 9.60 | 6.48-13.2 |
| Vertebra | T-transverse pr. | 24 | 70 | 3.36 | 0.72-7.92 | 20.88 | 7.92-46.08 | 5.76 | 2.88-10.32 |

Table S2.6 Segment-specific volumes for 15- year-old female

| Site | Segment | *n_s_×k_s_* | Voxel resolution | volume, cm^3^ | | | | | |
| --- | --- | --- | --- | --- | --- | --- | --- | --- | --- |
|  |  |  |  | TBV | | BM | | CBV | |
|  |  |  |  | reference | 90% CI | reference | 90% CI | reference | 90% CI |
| Clavicle | Acromial end | 2 | 130 | 1.10 | 0.42-2.42 | 2.74 | 1.6-4.94 | 1.20 | 0.76-1.88 |
| Clavicle | Shaft | 2×3.75 | 100 | 1.05 | 0.6-1.95 | 6.15 | 3.6-10.35 | 10.50 | 6.23-11.85 |
| Clavicle | Sternal end | 2 | 100 | 2.20 | 0.82-2.44 | 5.22 | 2.72-6.92 | 1.56 | 0.9-2.12 |
| Femur | Neck | 2 | 140 | 8.62 | 4.44-13.74 | 15.84 | 11.1-23.14 | 9.24 | 7.18-13.2 |
| Femur | Trochanter area | 2 | 150 | 14.54 | 8.78-17.8 | 44.74 | 29.26-60.32 | 17.46 | 13.06-19.24 |
| Humeri | Proximal end | 2 | 140 | 9.64 | 4.6-12.56 | 36.72 | 26.06-53.2 | 5.90 | 3.86-7.22 |
| Pelvis | Acetabulum | 2 | 110 | 7.50 | 0.34-18.72 | 22.50 | 0.88-55.32 | 12.90 | 6.02-21 |
| Pelvis | Iliac ala | 2×2.39 | 110 | 8.60 | 5.83-14.53 | 26.72 | 20.6-36.61 | 5.45 | 4.88-6.69 |
| Pelvis | Iliac crest | 2×4.93 | 100 | 8.09 | 4.54-10.45 | 24.45 | 17.16-38.36 | 9.76 | 7.3-12.23 |
| Pelvis | Iliac dorsal segment | 2×3.73 | 110 | 28.86 | 22.35-34.93 | 87.54 | 74.37-117.36 | 10.58 | 9.18-13.1 |
| Pelvis | Ischium ramus | 2×2.61 | 110 | 23.14 | 16.12-25.38 | 72.18 | 48.62-94.64 | 8.89 | 5.72-13 |
| Pelvis | Pubis ramus inferior | 2 | 110 | 5.98 | 2.04-12.32 | 18.62 | 7.52-52.04 | 2.80 | 1.3-4.96 |
| Pelvis | Pubis ramus superior (low) | 2 | 110 | 5.78 | 2.66-7.72 | 18.38 | 10.5-26.74 | 4.42 | 3.26-6.26 |
| Pelvis | Pubis ramus superior (upper part) | 2 | 110 | 5.90 | 2.12-12.58 | 18.56 | 7.92-32.72 | 4.68 | 2.9-7.2 |
| Ribs | 1, 2 | 2×10.86 | 60 | 3.91 | 2.61-6.52 | 28.89 | 17.59-50.61 | 17.38 | 13.68-23.24 |
| Ribs | 11, 12 | 2×32.59 | 60 | 4.56 | 1.96-6.52 | 34.55 | 16.95-59.97 | 35.20 | 22.81-47.58 |
| Ribs | 3, 4, 9, 10 | 2×37.38 | 60 | 11.96 | 2.99-13.46 | 88.96 | 37.38-160.73 | 50.84 | 36.63-58.31 |
| Ribs | 5,6,7,8 | 2×9.62 | 60 | 4.03 | 1.34-3.84 | 29.57 | 18.62-45.89 | 15.17 | 10.18-15.74 |
| Sacrum | Body 1 | 1 | 75 | 3.10 | 1.07-6.18 | 19.53 | 13.58-32.7 | 2.55 | 1.97-3.28 |
| Sacrum | Body 2-3 | 1 | 75 | 1.98 | 0.77-2.76 | 12.64 | 6.04-16.09 | 2.85 | 1.51-3.37 |
| Sacrum | Body 4-5 | 1 | 75 | 0.82 | 0.43-1.94 | 5.28 | 3.1-8.54 | 2.20 | 1.53-3.78 |
| Sacrum | Pedicle 1 | 2 | 75 | 0.80 | 0.52-1.74 | 5.22 | 2.8-8.94 | 1.84 | 1.02-2.36 |
| Sacrum | Pedicle 2 | 2 | 75 | 0.72 | 0.32-1.32 | 4.68 | 1.8-8.74 | 1.82 | 0.72-2.54 |
| Sacrum | Pedicle 3 | 2 | 75 | 0.50 | 0.18-0.82 | 3.16 | 1.42-4.74 | 1.44 | 0.64-1.46 |
| Sacrum | Pedicle 4 | 2 | 75 | 0.30 | 0.08-0.78 | 1.92 | 0.78-3.82 | 1.14 | 0.62-2.18 |
| Sacrum | Sacral ala 1 | 2 | 75 | 5.68 | 2.86-7.74 | 36.00 | 20.7-57.94 | 7.00 | 5.74-9.68 |
| Sacrum | Sacral ala 2 | 2 | 75 | 3.28 | 1.68-7.42 | 19.98 | 13.12-33.74 | 3.98 | 2.9-5.32 |
| Sacrum | Sacral ala 3-4 | 2 | 75 | 1.22 | 0.36-2.24 | 7.86 | 3.58-14.4 | 3.72 | 2.02-5.16 |
| Scapula | Acromion | 2 | 180 | 2.28 | 0.94-5.7 | 8.56 | 3.96-14.46 | 3.56 | 3.04-4.38 |
| Scapula | Glenoid | 2 | 180 | 3.36 | 2.26-7.82 | 12.38 | 7.64-20.66 | 2.46 | 1.8-3.58 |
| Scapula | Lateral margin | 2×3.52 | 180 | 0.84 | 0.35-1.69 | 3.03 | 1.9-3.8 | 3.38 | 2.46-4.44 |
| Skull | Flat bones | 1×33.68 | 200 | 41.09 | 27.95-59.61 | 37.72 | 26.61-49.51 | 78.81 | 72.75-90.94 |
| Sternum | Sternum | 1×7.08 | 90 | 8.22 | 4.11-13.03 | 47.88 | 34.14-60.63 | 11.47 | 8.57-15.44 |
| Vertebra | C1 mass | 2 | 60 | 0.72 | 0.28-1.26 | 2.68 | 1.44-4.94 | 2.54 | 1.84-3.72 |
| Vertebra | C2-body | 1 | 60 | 0.96 | 0.61-1.3 | 3.64 | 2.76-5.65 | 0.20 | 0.17-0.26 |
| Vertebra | C3-7 body | 5 | 100 | 2.60 | 0.8-3.45 | 9.95 | 6.75-13.4 | 0.95 | 0.7-1.2 |
| Vertebra | L- body | 5 | 70 | 17.50 | 10.65-23.5 | 112.65 | 68-139.4 | 5.85 | 3.05-6 |
| Vertebra | L-lamina+inf.pr. | 10 | 75 | 0.70 | 0.2-1.8 | 4.30 | 1.5-11 | 5.70 | 2.5-13.2 |
| Vertebra | L-spinous pr. | 5 | 70 | 2.35 | 0.8-3 | 14.50 | 9.3-21 | 4.00 | 2.85-4.4 |
| Vertebra | L-superior pr. | 10 | 50 | 2.90 | 1.2-3.5 | 18.70 | 10.1-21.4 | 3.60 | 1.7-4.4 |
| Vertebra | L-transverse pr. | 10 | 70 | 1.30 | 0.6-2.5 | 8.10 | 5.2-12.2 | 2.90 | 2.2-3.8 |
| Vertebra | T- body | 12 | 70 | 16.56 | 6.36-27.96 | 101.40 | 59.64-192.84 | 7.44 | 3.84-10.32 |
| Vertebra | T- lamina+inf. pr. | 24 | 60 | 1.68 | 0.24-3.84 | 11.04 | 1.2-29.04 | 20.16 | 11.04-29.28 |
| Vertebra | T-spinous pr. | 12 | 70 | 1.80 | 0.6-2.16 | 11.40 | 4.68-19.32 | 4.56 | 2.52-7.08 |
| Vertebra | T-superior pr. | 24 | 60 | 0.48 | 0.24-1.2 | 3.36 | 1.2-6.24 | 9.60 | 6.48-13.2 |
| Vertebra | T-transverse pr. | 24 | 70 | 3.36 | 0.72-7.92 | 20.88 | 7.92-46.08 | 5.76 | 2.88-10.32 |

Table S2.7 Segment-specific volumes for adult male

| Site | Segment | *n_s_×k_s_* | Voxel resolution | volume, cm^3^ | | | | | |
| --- | --- | --- | --- | --- | --- | --- | --- | --- | --- |
|  |  |  |  | TBV | | BM | | CBV | |
|  |  |  |  | reference | 90% CI | reference | 90% CI | reference | 90% CI |
| Clavicle | Body acromial end | 2 | 100 | 2.58 | 1.14-6.1 | 19.00 | 13.18-44.66 | 8.84 | 4.58-17.38 |
| Clavicle | Body sternal end | 2 | 100 | 1.34 | 1.06-2.76 | 9.74 | 6.38-24.32 | 6.86 | 2.82-11.7 |
| Clavicle | Ends | 4 | 90 | 10.04 | 3.88-11.16 | 25.24 | 13.96-36.08 | 3.92 | 2.28-4.52 |
| Femur | Neck | 2 | 100 | 7.36 | 5.58-10.46 | 36.86 | 27.18-47.6 | 11.88 | 9.82-15.34 |
| Femur | Trochanter area | 2 | 70 | 10.62 | 6.94-18.74 | 87.26 | 63.48-156.3 | 25.00 | 12.9-53.5 |
| Humeri | Proximal end | 2 | 70 | 4.04 | 0.7-7.56 | 64.52 | 39.18-83.92 | 6.94 | 4.58-9.1 |
| Pelvis | Acetabulum | 2 | 80 | 5.38 | 1.5-7.72 | 24.56 | 9.54-42.52 | 12.96 | 9.2-15.74 |
| Pelvis | Iliac ala | 2×3.7 | 60 | 9.03 | 2.66-12.8 | 40.92 | 14.8-49.51 | 13.17 | 9.99-16.8 |
| Pelvis | Iliac crest | 2×5.37 | 80 | 6.55 | 2.36-9.45 | 28.78 | 15.57-43.17 | 11.06 | 8.16-11.92 |
| Pelvis | Iliac dorsal segment | 2×2.4 | 80 | 13.20 | 9.98-22.18 | 60.10 | 51.46-80.59 | 8.98 | 5.81-10.85 |
| Pelvis | Ischium ramus | 2×1.83 | 140 | 25.55 | 16.76-38.43 | 82.46 | 55.52-130.15 | 9.33 | 6.08-13.14 |
| Pelvis | Pubis ramus inferior | 2 | 140 | 5.76 | 2.64-11.3 | 18.58 | 7.48-44.04 | 3.10 | 1.18-4.32 |
| Pelvis | Pubis ramus superior (low) | 2 | 140 | 3.98 | 1.08-9.16 | 19.50 | 7.62-56.38 | 4.36 | 2.26-7.76 |
| Pelvis | Pubis ramus superior (upper part) | 2 | 140 | 3.36 | 0.78-6.86 | 16.70 | 4.28-37.14 | 3.76 | 1.72-6.02 |
| Ribs | 1,2 | 2×12.7 | 60 | 7.87 | 3.81-14.48 | 57.66 | 39.12-92.71 | 25.40 | 19.05-27.69 |
| Ribs | 11, 12 | 2×12.6 | 60 | 3.78 | 2.02-7.31 | 28.98 | 17.39-44.6 | 16.88 | 12.6-21.67 |
| Ribs | 3, 4, 9, 10 | 2×39.4 | 60 | 18.12 | 7.09-24.43 | 132.38 | 79.59-139.48 | 65.40 | 46.49-75.65 |
| Ribs | 5,6,7,8 | 2×43.4 | 60 | 25.17 | 8.68-39.06 | 188.36 | 116.31-294.25 | 77.25 | 55.55-85.93 |
| Sacrum | Body 1 | 1 | 70 | 3.84 | 1.66-6.67 | 21.87 | 13.74-40.15 | 3.70 | 2.82-4.91 |
| Sacrum | Body 2-3 | 1 | 70 | 2.37 | 1.2-3.23 | 13.43 | 9.41-28.13 | 3.97 | 3.04-6.47 |
| Sacrum | Body 4-5 | 1 | 70 | 0.75 | 0.23-1.04 | 4.25 | 2-6.01 | 3.03 | 2.05-3.7 |
| Sacrum | Pedicle 1 | 2 | 70 | 0.82 | 0.34-1.08 | 4.72 | 2.82-8 | 2.40 | 1.58-3.28 |
| Sacrum | Pedicle 2 | 2 | 70 | 0.76 | 0.44-1.32 | 4.40 | 2.46-9.18 | 2.42 | 1.82-3.96 |
| Sacrum | Pedicle 3 | 2 | 70 | 0.50 | 0.18-1.1 | 2.88 | 1.26-6.4 | 1.90 | 1.08-3.34 |
| Sacrum | Pedicle 4 | 2 | 70 | 0.30 | 0.12-0.54 | 1.74 | 0.74-3.16 | 1.50 | 0.92-2.12 |
| Sacrum | Sacral ala 1 | 2 | 70 | 6.12 | 2.82-7.54 | 34.88 | 20.32-61.46 | 9.52 | 6.82-13.24 |
| Sacrum | Sacral ala 2 | 2 | 70 | 3.60 | 1.32-6.34 | 20.86 | 8.14-46.32 | 5.44 | 3-9.24 |
| Sacrum | Sacral ala 3-4 | 2 | 70 | 1.36 | 0.58-2.84 | 7.70 | 4.16-11.32 | 3.70 | 2.56-4.36 |
| Scapula | Acromion | 2 | 70 | 2.50 | 0.52-3.96 | 14.14 | 4.36-27.36 | 5.36 | 2.68-7.68 |
| Scapula | Glenoid | 2 | 70 | 3.82 | 1.88-6.02 | 21.76 | 14.76-32.38 | 3.86 | 2.32-5.44 |
| Scapula | Lateral margin | 2×3.9 | 70 | 0.94 | 0.39-1.09 | 4.99 | 2.42-6.55 | 4.91 | 3.82-5.69 |
| Skull | Flat bones | 2×37.7 | 100 | 59.94 | 20.74-107.07 | 55.42 | 33.55-127.05 | 95.00 | 74.65-101.79 |
| Sternum | Body | 1×7.08 | 110 | 7.22 | 4.18-9.91 | 42.55 | 37.67-48.99 | 13.74 | 10.34-15.43 |
| Sternum | Manubrium | 1×3.2 | 110 | 4.19 | 2.02-5.66 | 24.77 | 19.3-28.35 | 8.29 | 6.72-9.57 |
| Vertebra | C1 mass | 2 | 60 | 0.72 | 0.28-1.26 | 2.68 | 1.44-4.94 | 2.54 | 1.84-3.72 |
| Vertebra | C2-body | 1 | 60 | 0.96 | 0.61-1.3 | 3.64 | 2.76-5.65 | 0.20 | 0.17-0.26 |
| Vertebra | C3-7 body | 5 | 60 | 3.00 | 1.6-4.7 | 11.45 | 4.7-16.5 | 1.05 | 0.6-1.25 |
| Vertebra | L- body | 5 | 50 | 24.80 | 12.15-27.95 | 142.75 | 91-190.85 | 6.85 | 2.7-8.25 |
| Vertebra | L-lamina+inf.pr. | 10 | 75 | 0.70 | 0.24-1.8 | 4.30 | 1.5-10.9 | 5.70 | 2.5-13.2 |
| Vertebra | L-spinous pr. | 5 | 50 | 2.35 | 0.4-3.75 | 16.10 | 3.45-23.9 | 3.85 | 1.35-5.9 |
| Vertebra | L-superior pr. | 10 | 50 | 2.90 | 1.2-3.5 | 18.70 | 10.1-21.4 | 3.60 | 1.7-4.4 |
| Vertebra | L-transverse pr. | 10 | 50 | 2.50 | 1.6-3.7 | 15.80 | 9.9-23.2 | 3.90 | 2.3-5.1 |
| Vertebra | T- body | 12 | 70 | 36.48 | 22.2-52.44 | 186.60 | 119.64-287.52 | 12.24 | 9.36-17.76 |
| Vertebra | T- lamina+inf. pr. | 24 | 60 | 1.68 | 0.24-3.84 | 11.04 | 1.2-29.04 | 20.16 | 11.04-29.28 |
| Vertebra | T-spinous pr. | 12 | 60 | 0.96 | 0.12-6 | 6.60 | 1.32-29.04 | 15.36 | 10.2-25.56 |
| Vertebra | T-superior pr. | 24 | 60 | 0.48 | 0.24-1.2 | 3.36 | 1.2-6.24 | 9.60 | 6.48-13.2 |
| Vertebra | T-transverse pr. | 24 | 60 | 4.32 | 2.16-10.8 | 29.76 | 18.72-51.6 | 21.12 | 12-29.76 |

Table S2.8 Segment-specific volumes for adult female

| Site | Segment | *n_s_×k_s_* | Voxel resolution | volume, cm^3^ | | | | | |
| --- | --- | --- | --- | --- | --- | --- | --- | --- | --- |
|  |  |  |  | TBV | | BM | | CBV | |
|  |  |  |  | reference | 90% CI | reference | 90% CI | reference | 90% CI |
| Clavicle | Body acromial end | 2 | 100 | 1.98 | 0.88-4.66 | 13.22 | 9.18-31.08 | 7.16 | 3.7-14.08 |
| Clavicle | Body sternal end | 2 | 100 | 1.06 | 0.84-2.18 | 7.18 | 4.7-17.92 | 5.70 | 2.34-9.72 |
| Clavicle | Ends | 4 | 90 | 8.16 | 3.16-9.08 | 20.00 | 11.04-28.56 | 3.52 | 2.04-4.04 |
| Femur | Neck | 2 | 100 | 4.28 | 2.86-6.74 | 20.78 | 14.22-38.1 | 9.16 | 6.3-12.04 |
| Femur | Trochanter area | 2 | 70 | 6.58 | 3-8.4 | 53.18 | 32.1-75.38 | 17.64 | 8.06-20.24 |
| Humeri | Proximal end | 2 | 70 | 4.08 | 0.7-7.64 | 63.96 | 38.84-83.18 | 7.62 | 5.04-10 |
| Pelvis | Acetabulum | 2 | 80 | 5.38 | 1.5-7.72 | 24.56 | 9.54-42.52 | 12.96 | 9.2-15.74 |
| Pelvis | Iliac ala | 2×3.7 | 60 | 9.03 | 2.66-12.8 | 40.92 | 14.8-49.51 | 13.17 | 9.99-16.8 |
| Pelvis | Iliac crest | 2×4.97 | 80 | 6.06 | 2.19-8.75 | 26.64 | 14.41-39.96 | 10.24 | 7.55-11.03 |
| Pelvis | Iliac dorsal segment | 2×2.4 | 80 | 13.20 | 9.98-22.18 | 60.10 | 51.46-80.59 | 8.98 | 5.81-10.85 |
| Pelvis | Ischium ramus | 2×2.6 | 140 | 42.64 | 27.98-64.12 | 208.05 | 140.09-328.38 | 20.07 | 13.1-28.29 |
| Pelvis | Pubis ramus inferior | 2 | 140 | 5.76 | 2.64-11.3 | 18.58 | 7.48-44.04 | 3.10 | 1.18-4.32 |
| Pelvis | Pubis ramus superior (low) | 2 | 140 | 4.00 | 1.08-9.2 | 19.50 | 7.62-56.4 | 5.14 | 2.66-9.14 |
| Pelvis | Pubis ramus superior (upper part) | 2 | 140 | 3.88 | 0.9-7.94 | 18.96 | 4.86-42.18 | 6.36 | 2.92-10.2 |
| Ribs | 1,2 | 2×10.9 | 60 | 3.92 | 2.62-6.54 | 28.99 | 17.66-50.79 | 17.44 | 13.73-23.33 |
| Ribs | 11, 12 | 2×9.6 | 60 | 1.34 | 0.58-1.92 | 10.18 | 4.99-17.66 | 10.37 | 6.72-14.02 |
| Ribs | 3, 4, 9, 10 | 2×37.4 | 60 | 11.97 | 2.99-13.46 | 89.01 | 37.4-160.82 | 50.86 | 36.65-58.34 |
| Ribs | 5,6,7,8 | 2×32.6 | 60 | 13.69 | 4.56-13.04 | 100.41 | 63.24-155.83 | 51.51 | 34.56-53.46 |
| Sacrum | Body 1 | 1 | 70 | 3.24 | 1.4-5.63 | 18.37 | 11.54-33.73 | 3.47 | 2.64-4.6 |
| Sacrum | Body 2-3 | 1 | 70 | 3.13 | 1.59-4.27 | 17.74 | 12.43-37.16 | 4.24 | 3.25-6.91 |
| Sacrum | Body 4-5 | 1 | 70 | 0.79 | 0.24-1.1 | 4.48 | 2.11-6.34 | 2.94 | 1.99-3.59 |
| Sacrum | Pedicle 1 | 2 | 70 | 0.82 | 0.34-1.08 | 4.72 | 2.82-8 | 2.40 | 1.58-3.28 |
| Sacrum | Pedicle 2 | 2 | 70 | 0.76 | 0.44-1.32 | 4.40 | 2.46-9.18 | 2.42 | 1.82-3.96 |
| Sacrum | Pedicle 3 | 2 | 70 | 0.50 | 0.18-1.1 | 2.88 | 1.26-6.4 | 1.90 | 1.08-3.34 |
| Sacrum | Pedicle 4 | 2 | 70 | 0.30 | 0.12-0.54 | 1.74 | 0.74-3.16 | 1.50 | 0.92-2.12 |
| Sacrum | Sacral ala 1 | 2 | 70 | 5.90 | 2.72-7.28 | 33.44 | 19.48-58.92 | 9.16 | 6.56-12.74 |
| Sacrum | Sacral ala 2 | 2 | 70 | 3.34 | 1.22-5.88 | 18.96 | 7.4-42.08 | 5.22 | 2.88-8.86 |
| Sacrum | Sacral ala 3-4 | 2 | 70 | 1.36 | 0.58-2.84 | 7.70 | 4.16-11.32 | 3.70 | 2.56-4.36 |
| Scapula | Acromion | 2 | 70 | 2.50 | 0.52-3.96 | 14.14 | 4.36-27.36 | 5.36 | 2.68-7.68 |
| Scapula | Glenoid | 2 | 70 | 3.82 | 1.88-6.02 | 21.76 | 14.76-32.38 | 3.86 | 2.32-5.44 |
| Scapula | Lateral margin | 2×3.9 | 70 | 0.94 | 0.39-1.09 | 4.99 | 2.42-6.55 | 4.91 | 3.82-5.69 |
| Skull | Flat bones | 2×33.7 | 100 | 53.58 | 18.54-95.71 | 49.54 | 29.99-113.57 | 84.92 | 66.73-90.99 |
| Sternum | Body | 1×2.9 | 110 | 2.38 | 1.27-3.48 | 14.23 | 11.04-17.85 | 5.35 | 2.67-7.51 |
| Sternum | Manubrium | 1×2.7 | 110 | 2.81 | 1.94-4.16 | 17.01 | 12.5-21.36 | 6.97 | 5.35-8.56 |
| Vertebra | C1 mass | 2 | 60 | 0.72 | 0.28-1.26 | 2.68 | 1.44-4.94 | 2.54 | 1.84-3.72 |
| Vertebra | C2-body | 1 | 60 | 0.96 | 0.61-1.3 | 3.64 | 2.76-5.65 | 0.20 | 0.17-0.26 |
| Vertebra | C3-7 body | 5 | 60 | 2.30 | 1.25-3.6 | 8.20 | 3.35-11.8 | 0.85 | 0.5-1 |
| Vertebra | L- body | 5 | 50 | 20.95 | 10.25-23.6 | 118.65 | 75.65-158.65 | 6.30 | 2.5-7.6 |
| Vertebra | L-lamina+inf.pr. | 10 | 75 | 0.70 | 0.24-1.8 | 4.30 | 1.5-10.9 | 5.70 | 2.5-13.2 |
| Vertebra | L-spinous pr. | 5 | 50 | 2.00 | 0.35-3.15 | 13.30 | 2.85-19.75 | 3.30 | 1.15-5.1 |
| Vertebra | L-superior pr. | 10 | 50 | 2.90 | 1.2-3.5 | 18.70 | 10.1-21.4 | 3.60 | 1.7-4.4 |
| Vertebra | L-transverse pr. | 10 | 50 | 2.50 | 1.6-3.7 | 15.80 | 9.9-23.2 | 3.90 | 2.3-5.1 |
| Vertebra | T- body | 12 | 70 | 23.52 | 14.4-33.84 | 123.72 | 79.32-190.68 | 9.00 | 6.84-13.08 |
| Vertebra | T- lamina+inf. pr. | 24 | 60 | 1.68 | 0.24-3.84 | 11.04 | 1.2-29.04 | 20.16 | 11.04-29.28 |
| Vertebra | T-spinous pr. | 12 | 60 | 0.96 | 0.12-6 | 6.60 | 1.32-29.04 | 15.36 | 10.2-25.56 |
| Vertebra | T-superior pr. | 24 | 60 | 0.48 | 0.24-1.2 | 3.36 | 1.2-6.24 | 9.60 | 6.48-13.2 |
| Vertebra | T-transverse pr. | 24 | 60 | 4.32 | 2.16-10.8 | 29.76 | 18.72-51.6 | 21.12 | 12-29.76 |
