# Supplementary material for: Stochastic parametric skeletal dosimetry model for humans: Pediatric and adult computational skeleton phantoms for internal bone marrow dosimetry
Source: PLoS One. 2025 Jul 3;20(7):e0327479. doi: 10.1371/journal.pone.0327479 (PMC12225816; doi:10.1371/journal.pone.0327479)
Supplement: Attachment — Submitted filename: PONE-D-25-02823r1.pdf [file pone.0327479.s005.pdf]

RESULTS

1. “The chemical composition” in line 234

- Isn't that the composition of the elements? Is information about the composition of the nuclides unnecessary?
- A: The chemical composition of media is a necessary characteristic of the computational phantom and is therefore included in our paper. In contrast, the radionuclide composition (and distribution) are variables, which could be set in calculation with the phantoms and is not the topic of our paper.
- It seems that the table 3 shows element composition not chemical composition. The (non-radio) nuclide composition such as Mg-24, Mg-25 and Mg-26 is critical for assessing the neutron dose from a high-energy alpha emitter.

2. Fig 6

- The slopes are shown in the line graph, so it might be good to indicate that Adults are 20 years old.
- A: Parameters of adult SPSD phantoms are based on results of morphometry measurements of specimens with ages greater than 18 years. It would be incorrect to say that adults are 20-years old.
- The horizontal axis is an ordinal scale, but is it fair to show the slope?
