# Supplementary material for: Stochastic parametric skeletal dosimetry model for humans: Pediatric and adult computational skeleton phantoms for internal bone marrow dosimetry
Source: PLoS One. 2025 Jul 3;20(7):e0327479. doi: 10.1371/journal.pone.0327479 (PMC12225816; doi:10.1371/journal.pone.0327479)
Supplement: Attachment — Submitted filename: PONE-D-25-02823.docx [file pone.0327479.s003.docx]

# General comment

This is a study on improving the dose evaluation model to avoid underestimating or overestimating the dose of beta rays to the bone marrow in internal exposure in typical individuals. It is thought that the study is also trying to answer questions from the public(KOBAYASHI 2019)(Kobayashi 2024).

Since underestimating the dose to the bone marrow leads to underestimating the risk for the exposed individual, and overestimating the dose leads to underestimating the risk in epidemiological studies, it would be better if the study also mentioned the impact it would have on radiation protection.

From the perspective of promoting the significance of the research, why not also explain the development from Reference 28?

# Specific comments

Introduction

## 1.  “modeling of a reference man” in line 79

- The term “a reference man” is now being superseded by the more general concept of the “reference individual”^[1]^.

## 2. “Another problem is a large error in cortical thickness values” in line 90

- The definition of “error” should be indicated regarding radiation protection.

## 3. “best calculate” on line 96

## Since “best” is a goal that cannot be reached, why not describe what is still lacking compared to the required performance?

- MATERIALS AND METHODS

## 4. “active red bone marrow; AM” in line 120

- Since its first appearance, “active red bone marrow” has been written as “active marrow”. This is probably to reduce redundancy.

RESULTS

## 5. Table 2

## Why not explain what the symbol “-” means?

## 6. “The chemical composition” in line 234

- Isn't that the composition of the elements? Is information about the composition of the nuclides unnecessary?
- “coefficients of variation (CVs)” in line 258
- In this paper, there is a mixture of percentages and non-percentages.

## 7. Table 7

- Is BV/TV in arbitrary units? BV and TV each have their own unit of volume, so isn't BV/TV showing a ratio?

## 8. Fig 5 “The error bars correspond to ± one standard deviation.”

- In “tibiae and fibulae”, the bone mass is close to 0 when it deviates by two standard deviations. The authors may wish to consider the characteristics of the distribution and indicate a more meaningful interval from the perspective of radiation protection.
- Fig 6
- The slopes are shown in the line graph, so it might be good to indicate that Adults are 20 years old.

DISCUSSION

## 9. “… for improved dosimetric modeling.” in line 341

- It would be better to explain the significance of this improvement in terms of radiation protection.

## 10. “65.3±4%” in line 345

- It would be good to explain what the ± symbol indicates.

## 11. Fig 7

- Is it intentional that the font changes after “and b)?” in the title.

## 12. “within the error of individual variability” in line 377

- Is it okay that it says “error”?

## 13. “nuclear magnetic resonance investigations” in line 386

- The notation is inconsistent with line 125.

## 14. Table 15

- The range of the SSPD for Clavicles is indicated by a hyphen-minus.

## 15. “Segment-specific dose factors” in line 432

- Why not make it clear that it is written as DF?

Ref.

KOBAYASHI, Noriko. 2019. “Evaluation of Doses Absorbed to a Bone Marrow Stem Cell Layer from Short-Lived Radionuclides in the Blood Vessels and from Long-Lived Radionuclides in the Cortical Bone.” *Japanese Journal of Health Physics* 54 (1). 55–65. doi:10.5453/jhps.54.55.

Kobayashi, Noriko. 2024. “Assessment of Absorbed Dose in the Hematopoietic Stem Cell Layer of the Bone Marrow, Assuming Non-Uniform Distribution around the Vascular Endothelium of the Bone Marrow.” doi:10.1101/2024.10.19.24315800.

1. https://vocabulary.iaea.org/iaea-safety-glossary/483.html
