# Supplementary material for: Stochastic parametric skeletal dosimetry model for humans: Pediatric and adult computational skeleton phantoms for internal bone marrow dosimetry
Source: PLoS One. 2025 Jul 3;20(7):e0327479. doi: 10.1371/journal.pone.0327479 (PMC12225816; doi:10.1371/journal.pone.0327479)
Supplement: Attachment — Submitted filename: Response to Reviewers 20250518.docx [file pone.0327479.s004.docx]

# Thank you for your comments and suggestions, which are very useful and important for improving our work. Below, our responses to your suggestions are marked with a bold letter **A** and phrases added to the manuscript are marked below in **bold**.

# General comment:

# This is a study on improving the dose evaluation model to avoid underestimating or overestimating the dose of beta rays to the bone marrow in internal exposure in typical individuals. It is thought that the study is also trying to answer questions from the public(KOBAYASHI 2019)(Kobayashi 2024).

Since underestimating the dose to the bone marrow leads to underestimating the risk for the exposed individual, and overestimating the dose leads to underestimating the risk in epidemiological studies, it would be better if the study also mentioned the impact it would have on radiation protection.

From the perspective of promoting the significance of the research, why not also explain the development from Reference 28?

**A**: Thanks for the suggestion, we have added the following sentence to the introduction:

**“Unbiased dose estimates are a critical component of performing radiation risk evaluations in epidemiological studies of an exposed population. Therefore, the computational phantoms will be useful in terms of radiological protection. The importance of these issues is emphasized, for example, in the works of Kobayashi [23,24].”**

# Specific comments:

Introduction

## 1. “modeling of a reference man” in line 79

- The term “a reference man” is now being superseded by the more general concept of the “reference individual”^[1]^.

**A**: **“Reference man” was changed to “reference individual”** except in cases where it specifically refers to the ICRP phantom (because it’s the phantom name).

## 2.  “Another problem is a large error in cortical thickness values” in line 90

The definition of “error” should be indicated regarding radiation protection.

**A**: Sentence was changed to **“Another** **problem** **is the biased** **estimation of cortical thickness values.”**

## 3. “best calculate” on line 96

## Since “best” is a goal that cannot be reached, why not describe what is still lacking compared to the required performance?

**A**: Sentence was changed to **“To calculate the doses more correctly, it is necessary to find ways to avoid the problems described above, especially regarding the doses in AM exposed to beta-emitters (such as ^89,90^Sr).”**

- MATERIALS AND METHODS

## 4. “active red bone marrow; AM” in line 120

Since its first appearance, “active red bone marrow” has been written as “active marrow”. This is probably to reduce redundancy.

**A**: “active red bone marrow” was corrected to **“hematopoietically active marrow”.** Here, the use of hematopoietically is equivalent to red.

RESULTS

## 5. Table 2

## Why not explain what the symbol “-” means?

**A**: **“† “-” indicates there is no AM for this age group and location”**, an explanation was added below the table.

## 6. “The chemical composition” in line 234

Isn't that the composition of the elements? Is information about the composition of the nuclides unnecessary?

**A**: The chemical composition of media is a necessary characteristic of the computational phantom and is therefore included in our paper. In contrast, the radionuclide composition (and distribution) are variables, which could be set in calculation with the phantoms and is not the topic of our paper.

“coefficients of variation (CVs)” in line 258

In this paper, there is a mixture of percentages and non-percentages.

**A**: CV values were corrected to percentages

## 7. Table 7

Is BV/TV in arbitrary units? BV and TV each have their own unit of volume, so isn't BV/TV showing a ratio?

**A**: “Arbitrary” units were corrected to **“relative”**

## 8. Fig 5 “The error bars correspond to ± one standard deviation.”

In “tibiae and fibulae”, the bone mass is close to 0 when it deviates by two standard deviations. The authors may wish to consider the characteristics of the distribution and indicate a more meaningful interval from the perspective of radiation protection.

**A**: Thank you for noticing, the error bars reflected 95% CI for SPSD phantoms data, and we have now updated the figure with standard deviation error bars.

Fig 6

The slopes are shown in the line graph, so it might be good to indicate that Adults are 20 years old.

**A**: Parameters of adult SPSD phantoms are based on results of morphometry measurements of specimens with ages greater than 18 years. It would be incorrect to say that adults are 20-years old.

DISCUSSION

## 9. “… for improved dosimetric modeling.” in line 341

It would be better to explain the significance of this improvement in terms of radiation protection.

**A**: Thank you for the suggestion. An explanation was added to the discussion. “**Our work to define bone geometry and bone segment parameters resulted in the development of phantoms to improve the bone marrow dosimetry. AM is a critical organ of hematopoiesis, therefore AM dosimetry is extremely important for clarifying the risks of hematological radiation effects. The representative computational phantoms need to produce accurate estimates of AM internal doses.”**

## 10. “65.3±4%” in line 345

It would be good to explain what the ± symbol indicates.

**A**: The volume fractions were expressed in relative units and **“(mean±SD)”** was added.

## 11. Fig 7

Is it intentional that the font changes after “and b)?” in the title.

**A**: Font was corrected

## 12. “within the error of individual variability” in line 377

Is it okay that it says “error”?

A: Thank you for noticing. “Error” was deleted

## 13. “nuclear magnetic resonance investigations” in line 386

The notation is inconsistent with line 125.

**A**: Was changed to **“MRI-data”**

## 14. Table 15

The range of the SSPD for Clavicles is indicated by a hyphen-minus.

**A**: Hyphen-minus was changed to dash

## 15. “Segment-specific dose factors” in line 432

Why not make it clear that it is written as DF?

A: Explanation was added: “Segment-specific dose factors (**DFs**)”

A: References were added as follows:

Kobayashi N. Evaluation of doses absorbed to a bone marrow stem cell layer from short-lived radionuclides in the blood vessels and from long-lived radionuclides in the cortical bone. Hoken Butsuri (online). 2019, 54(1): 55–65. <https://doi.org/10.5453/jhps.54.55>

Kobayashi N. Assessment of absorbed dose in the hematopoietic stem cell layer of the bone marrow, assuming non-uniform distribution around the vascular endothelium of the bone marrow. medRxiv (online). 2024. <https://doi.org/10.1101/2024.10.19.24315800>.

1. https://vocabulary.iaea.org/iaea-safety-glossary/483.html
