# Supplementary material for: Stochastic parametric skeletal dosimetry model for humans: Pediatric and adult computational skeleton phantoms for internal bone marrow dosimetry
Source: PLoS One. 2025 Jul 3;20(7):e0327479. doi: 10.1371/journal.pone.0327479 (PMC12225816; doi:10.1371/journal.pone.0327479)
Supplement: Attachment — Submitted filename: Response to Reviewers 20250615.docx [file pone.0327479.s006.docx]

# Thank you for your comments and suggestions, it is very useful and important for our work. Below are the answers to your follow-up suggestions, marked with the letter "**B**". Note that previous responses are marked with the letter “A”. New text added to the manuscript is marked in **bold**. General comment

1. “The chemical composition” in line 234

• Isn't that the composition of the elements? Is information about the composition

of the nuclides unnecessary?

• A: The chemical composition of media is a necessary characteristic of the

computational phantom and is therefore included in our paper. In contrast, the

radionuclide composition (and distribution) are variables, which could be set in

calculation with the phantoms and is not the topic of our paper.

• It seems that the table 3 shows element composition not chemical composition.

The (non-radio) nuclide composition such as Mg-24, Mg-25 and Mg-26 is critical

for assessing the neutron dose from a high-energy alpha emitter.

**B**: We have made corrections in the manuscript; the term chemical composition has been replaced by **elemental composition**. As indicated in the Introduction (lines 97–108), phantoms are created for dosimetry of beta emitters only, so we did not include the isotopic composition in this paper.

2. Fig 6

• The slopes are shown in the line graph, so it might be good to indicate that

Adults are 20 years old.

• A: Parameters of adult SPSD phantoms are based on results of morphometry

measurements of specimens with ages greater than 18 years. It would be

incorrect to say that adults are 20-years old.

• The horizontal axis is an ordinal scale, but is it fair to show the slope?

**B**: The slope in Fig 6 reflects the tendency to increase the volume of phantoms, not the age dependence. Fig 6 and Fig 8 were corrected by adding breaks between 15-years-old and adults to the graphs.
